# Early Sri Lankan coastal site tracks technological change and estuarine resource exploitation over the last ca. 25,000 years

**DOI:** 10.1038/s41598-024-77504-5

**Published:** 2024-11-04

**Authors:** Noel Amano, Patrick Faulkner, Oshan Wedage, Chris Clarkson, Dambara Amila, Miren del Val, Dovydas Jurkenas, Alexander Kapukotuwa, Gloria I. López, Josep Pares, M. M. Pathmalal, Tam Smith, Martin Wright, Patrick Roberts, Michael Petraglia, Nicole Boivin

**Affiliations:** 1 Max Planck Institute of Geoanthropology, Jena, Germany; 2 School of Humanities, Faculty of Arts and Social Sciences, University of Sydney, Sydney, Australia; 3 Department of History and Archaeology, University of Sri Jayewardenepura, Gangodawila, Nugegoda Sri Lanka; 4 School of Social Sciences, University of Queensland, Brisbane, QLD Australia; 5 Spanish National Research Center for Human Evolution (CENIEH), Burgos, Spain; 6 Colombian Geological Society, Bogotá, Colombia; 7 Nuclear Affairs Directorate, Colombian Geological Survey, Bogotá, Colombia; 8 Department of Zoology, University of Sri Jayewardenepura, Gangodawila, Nugegoda Sri Lanka; 9 isoTROPIC Research Group, Max Planck Institute of Geoanthropology, Jena, Germany; 10 Australian Research Centre for Human Evolution, Griffith University, Brisbane, QLD Australia; 11 Human Origins Program, National Museum of Natural History, Smithsonian Institution, Washington, DC USA; 12 Griffith Sciences, Griffith University, Brisbane, QLD Australia; 13 Leon Recanati Institute for Maritime Studies, University of Haifa, Haifa, Israel

**Keywords:** Archaeology, Climate-change adaptation

## Abstract

The island of Sri Lanka was part of the South Asian mainland for the majority of the past 115,000 years, and connected most recently during the Last Glacial Maximum via the now submerged Palk Strait. The degree to which rising sea levels shaped past human adaptations from the Pleistocene and into the mid to late Holocene in Sri Lanka has remained unclear, in part because the earliest reliable records of human occupation come from the island’s interior, where cave sites have revealed occupation of tropical forest ecosystems extending back to 48 thousand years (ka). The island’s earliest known open-air sites are all much younger in date, with ages beginning at 15 ka and extending across the Holocene. Here we report the earliest well-dated open-air coastal site in Sri Lanka, Pathirajawela, which records human occupation back to ca. 25,000 years ago. We show that humans at Pathirajawela consistently adapted to changing ecosystems linked to sea level transgression and coastal evolution from the Last Glacial Maximum into the Holocene. The presence of anthropogenic shell midden deposits at the site from ca. 4.8 ka, focused almost exclusively on a single taxon, indicates intensification of estuarine resource exploitation, as humans responded to opportunities presented by the formation of new coastal ecosystems.

## Introduction

Sri Lanka, located between 5° and 10°N, ca. 50 km off the south-eastern edge of the Indian subcontinent, has produced some of the earliest evidence for modern human presence in southern Asia. Fossil reef^1^ and modelling of bathymetric data^2–6^ suggest that the island has been intermittently connected to mainland South Asia during periods of increased global aridity and low sea levels, most recently during the Last Glacial Maximum (LGM) with eustatic sea-level fall of ca. 130 m^7^ exposing a wide land bridge across the Palk Strait. This has important implications not only for biogeography but also understanding human adaptations to rising sea levels and changing coastlines following the LGM.

Archaeological investigations in Sri Lanka have contributed significantly to understanding the adaptations of human populations to the variable ecosystems that they encountered in the region. Archaeological sites in the island have provided some of the earliest direct evidence for human reliance on rainforest resources anywhere in the world^2,8–11^, challenging previously held views of tropical rainforests as ecological barriers to successful population movements^12,13^. These adaptations consist of specialised hunting of difficult-to-capture arboreal prey aided by microlithic^14^ and osseous tools^15^, including the use of bow-and-arrow technology^16^, as well as utilisation of nuts and fruits for food and plant fibre possibly for cordage and clothing^11^. Analyses of stable carbon and oxygen isotopes on human and faunal dental enamel (based on > 600 samples) showed heavy reliance on rainforest resources from the Late Pleistocene to the Mid-Holocene and year-long exploitation of these resources^8,9,17^. However, the long-term record of human occupation in Sri Lanka is primarily derived from three rock shelter and cave sites, located in the island’s western upland Wet Zone region: Batadomba-lena, dated between ca. 39–10 ka cal BP^9,18^, Fa-Hien Lena dated to ca. 48–4.2 ka cal BP^10^ and Kitulgala Beli-lena dated to ca. 45–8 ka cal BP^11^.

**Fig. 1:**
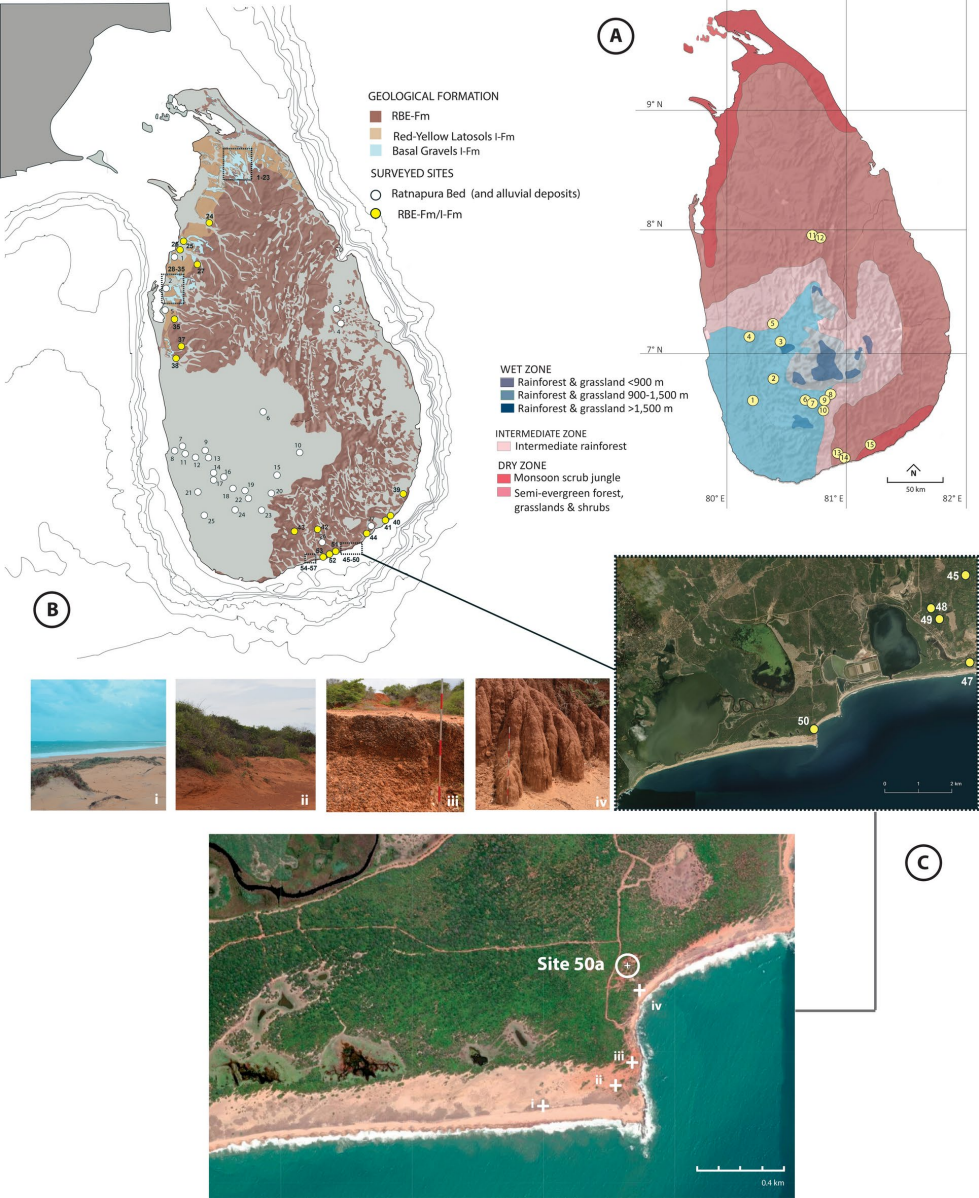
(**A**) Location of dated Late Pleistocene to Mid-Holocene sites in Sri Lanka (see TableS1) as well as the island’s vegetation zones (redrawn from Erdelen^67^ and Ashton and Gunatilleke^68^); (**B**) Location of sites surveyed by S. Deraniyagala including stone tool-bearing sites within the Iranamadu formation (redrawn from ref^21^); (**C**) Location of Pathirajawela (Site 50a) well as geological features around the site (i): sand dunes near the coast, (ii): surface of the stone-tool bearing red latosol layer, (iii): basal gravel layer underlying the red latosol in an exposed profile, (iv): ca. 2 m red latosol exposed profile. Maps throughout this article were created using ArcGIS Pro2.7 (2023) software by Esri. ArcGIS® is the intellectual property of Esri and are used herein under license. Copyright © Esri. All rights reserved. For more information about Esri® software, please visit www.esri.com.

Although there is evidence for the transportation and modification of marine shell c. 48,000–34,000 years ago to the inland sites of Fa-Hien Lena and Batadomba-lena^9,18^, the nature of contemporaneous or earlier records of human occupation on Sri Lanka’s coastlines is less well understood. Late Pleistocene to Mid-Holocene archaeological sites have been documented in Sri Lanka’s Intermediate and Dry Zones and on the southern semi-arid coast (Fig. 1A, Table SI 1). These include cave and rock shelter sites such as Balangoda Kuragala (ca. 15–5 ka cal BP)^8,19,20^, Udipiyan Galge (ca 9.5 ka cal BP)^21^, Potana (ca. 5.9–5.7 ka cal BP)^22^, Lunugal-ge (ca. 5.9 ka cal BP)^23^ and Alugal-ge (ca. 5.4 ka cal BP)^23^; open-air sites like Bellan-bandi Palassa (ca. 12.3–11.2 ka cal BP)^21,24^, and the shell midden sites of Kalamatiya (ca. 5–3.9 ka cal BP)^25^, Mini-athiliya (ca. 5–2.8 ka cal BP)^26^ and Pallemalala (ca. 4.7–2.5 cal BP)^25,27,28^. These sites yielded stone tools, specifically geometric microliths, as well as abundant faunal (both vertebrate and molluscan) and botanical remains. Human inhumations were also reported in some sites, notably from Bellan-bandi Palassa (ca. 12,000–11,000 cal BP)^8,29,30^, Balangoda Kuragala (ca 7000 cal BP)^8,19,20^ and Mini-athiliya (ca. 3600 cal BP)^25,26,31^. In the context of broader South Asian connections, such as the identification of near-coastal deposits dating to the Late Pleistocene in northwest India^32^ and greater accessibility through the Palk Strait from the LGM until the early to mid-Holocene^33^, the earliest evidence for coastal occupation and marine resource use would be expected to come from the northern and northwestern regions of Sri Lanka. At this stage, however, there are no known sites in these regions that date to the critical Late Pleistocene to Mid-Holocene window, with sites either being much more recent^34^ or remaining undated (Fig. 1B).

In addition to the dated sites noted above, possible Late Pleistocene to Mid-Holocene archaeological open-air sites have been identified through surveys and test trench excavations on the island’s southern coast^21^. Systematic excavation and dating of these sites, as well as comprehensive analyses of recovered archaeological materials, however, remain limited. One site in particular, Pathirajawela, was dated to ca. 70 ka, leading some scholars to suggest that the earliest human occupation sites in Sri Lanka were located along the coast^21^. Given the relative absence of well dated, stratified coastal and near-coastal archaeological sites in Sri Lanka, and more broadly across South Asia, detailed investigations of these open-air coastal sites would fill gaps in existing knowledge of the emergence of coastal adaptations in Sri Lanka and the way communities responded to shifting climatic and environmental conditions from the late Pleistocene onwards.

Here we report the results of the systematic re-excavation of the open-air coastal site of Pathirajawela, located in Sri Lanka’s Southern Province, offering new dates and the first systematic archaeological analysis of Sri Lanka’s earliest coastal site. We focus on the site’s chronostratigraphy, providing evidence for long-term human activities from 24.8–1.2 ka, a unique window spanning the period before and during major sea-level transgression. We describe changes in the lithic artefact sequence and as well as evidence for targeted utilisation of estuarine resources coinciding with the Mid- Holocene Sea level high stand. Our findings also highlight the potential for sites outside the Sri Lankan wet rainforest zone to provide important insights into long term human–environment interactions on the island, including adaptations to regional ecosystem transformation as the continental shelf was increasingly submerged.

### Excavation and site chronostratigraphy

Pathirajawela was first excavated by Siran Deraniyagala (as Site 50a) during his 1972 survey of tool-bearing sites within the Iranamadu Formation (IFm). First identified by EJ Wayland in 1915 and described in detail by Deraniyagala^21^, the IFm is an extensive stratigraphic unit positioned between the bedrock (variably basement gneisses, Jurassic sedimentaries and Tertiary rocks) and overlying Holocene deposits (alluvia, colluvia, soils and estuarine deposits) appearing on much of the coastal lowland of Sri Lanka, particularly in the island’s Dry Zone region (Fig. 1B). Although the IFm stratigraphy remains provisional, it has been consistently described as consisting of two distinct units: i) basal gravel (Fig. 1C-iii) composed of sub-angular to well-rounded particles, predominantly quartz, associated with ironstone nodules that tend to form both ferricrete conglomerate and ii) overlying lateritised aeolian sands (Fig. 1C-iv), similar to the extensive *teri* deposits in coastal Tamil Nadu, southern India^21,35^.

Deraniyagala’s survey and test excavations of IFm sites yielded stone artefacts from both IFm units, with apparent ‘Middle Palaeolithic-type tools’ (termed ‘Mousteroid’) from the basal gravel layers and flake-based and microlithic stone ‘tools’ from the aeolian sands. Thermoluminescence dating of the IFm provided ages of ca. 64.4–74.2 ka for the basal gravels and ca. 28.5 ka for the lateritised sand^36^. In many sites, especially on Sri Lanka’s southern coast, a distinct shell layer was deposited between the IFm formation and the overlying soils. The nature of this shell accumulation, ranging in thickness from ca. 5–50 cm, dated between ca. 5.5–3.4 ka BP and yielding cultural remains including human burials, remains a topic of discussion. Some researchers argue for anthropic origin (i.e. midden)^21^ whereas others suggest that the layer resulted from natural transport of shell deposited during sea-level transgression through wave action and storm surges (i.e., chenier)^25,37^.

**Fig. 2:**
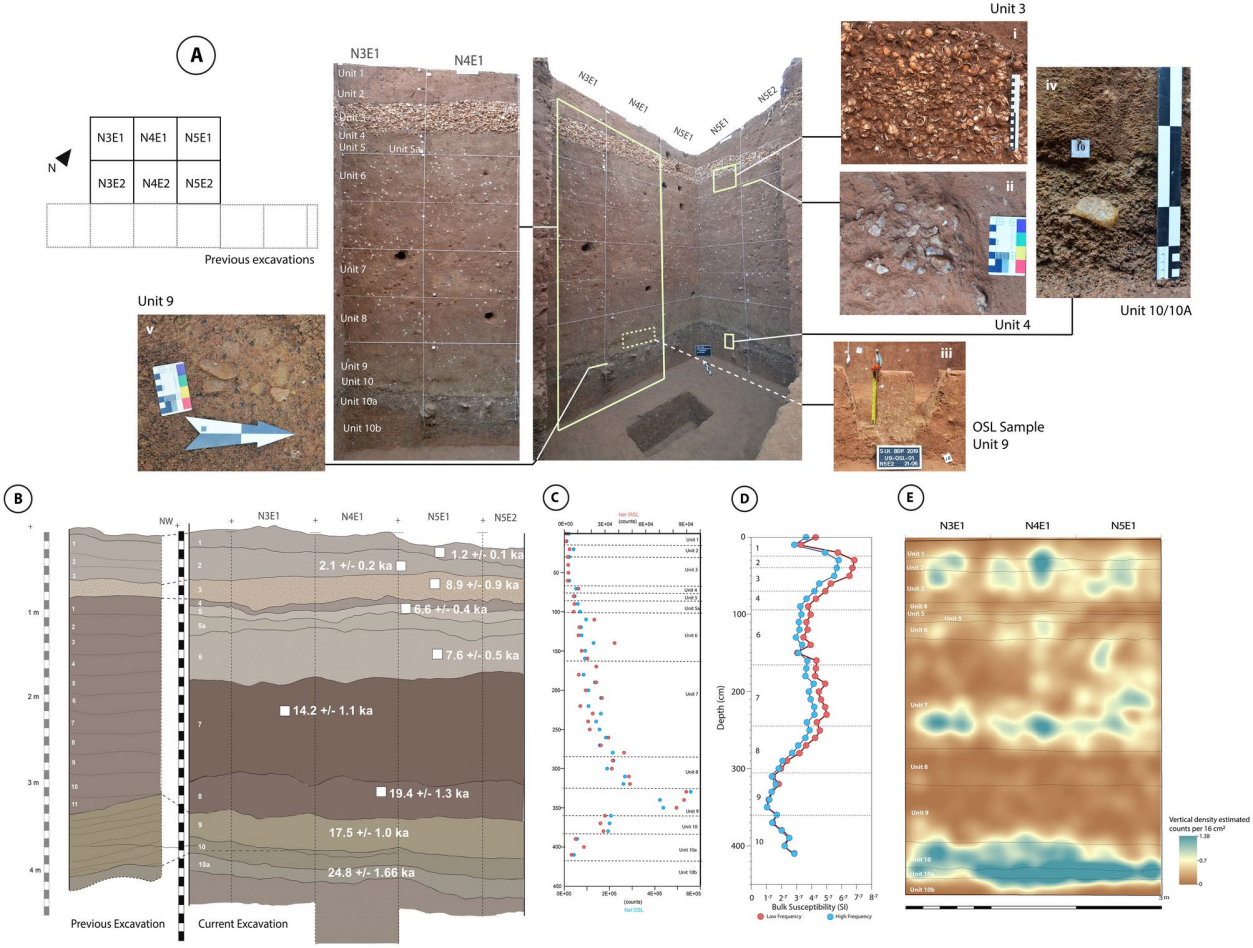
Stratigraphic profile of Pathirajawela showing the major archaeological units (**A**) and associated OSL dates (**B**) (see Table S3); (**C**) Luminescence profile showcasing OSL and IRSL values measured by POSL over the entire stratigraphic sequence; (**D**) Bulk magnetic susceptibility measurements of sediment samples from the stratigraphic sequence; (**E**) Kernel density estimation of the vertical distribution of lithic materials recovered from the site.

Pathirajawela (6°10′16″N 81°13′30″E) is located within Bundala National Park in Sri Lanka’s Southern Province at ca. 15–20 m ASL, approximately 200 m from the current shoreline (Fig. 1B–C). Following the grid of the 1970s excavation, our interdisciplinary archaeological team opened six 1m^2^ excavation squares in 2019, exposing a ca. 4.5 m stratigraphic profile, removing approximately 16.7 m^3^ of sediments (Fig. 2A; Table 1). Our re-excavation of Pathirajawela marks the start of a project that aims to reinvestigate and date the more than 50 archaeological sites currently known within the IFm formation. For Pathirajawela specifically, our study aimed to refine the site stratigraphy, provide new Optically Stimulated Luminescence (OSL) dates for the IFm deposit as well as the overlying soils, evaluate the stone artefact sequence via changes in techno-typology and raw materials, and contribute to the understanding of the nature of the shell-bearing layer. 

**Table 1:** Stratigraphic units identified in the excavation of Pathirajawela and total number and density of lithic materials recovered from each unit.

Archaeological unit	1970s Stratigraphy	Lithic artefacts (n)			Estimated sediment volume (m^3^)	Density per m^3^	Thickness (ca. cm)	Date (ka)
		Plotted	From sieving	Total				
1	Stratum V Level 1	126	419	545	0.808	674.50	0.2	1.2 ± 0.1
2	Stratum V Levels 2–3	154	456	610	0.552	1105.07	0.3	2.1 ± 0.2
3	Stratum IV Levels 1–2	275	328	603	1.48	407.43	0.2	8.9 ± 0.9
4	Stratum III Level 1	27	7	34	0.44	77.27	0.1	NA
5	Stratum III Level 2	20	1	21	0.122	172.13	0.1	6.6 ± 0.4
5a	Stratum III Level 2	42	115	157	0.381	412.07	0.2	NA
6	Stratum III Levels 3–4	108	135	243	0.795	305.66	0.5	7.6 ± 0.5
7	Stratum III Levels 5–8	858	727	1585	5.218	303.76	1.2	14.2 ± 1.1
8	Stratum III Levels 9–10	46	7	53	1.65	32.12	0.4	19.4 ± 1.3
9	Stratum III Level 11	100	4	104	2.728	38.12	0.4	17.5 ± 1.0
10	Stratum II Level 1	334	97	431	1.216	354.44	0.2	NA
10a	Stratum II Levels 2–3	339	6	345	0.433	796.77	0.2	24.8 ± 1.66
10b	Stratum II Level 4	88	48	136	0.865	157.23	0.4	NA

Our excavation identified a total of 13 archaeological units corresponding to the sediment strata described by the previous investigators (Fig. 2B; Table 1). Units 10b, 10a and 10 represent the lower pisolith-rich gravel member of the IFm. Unit 10b consists of matrix-poor, well sorted gravel with abundant Fe/Mn pisoliths, differentiated from the overlying Units 10a and 10 by having relatively larger particles (0.5–2 cm compared to 0.3–1 cm) and less rounded Fe/Mn oxide-encrusted quartz and gneiss clasts. Unit 10b overlies weathered and kaolinized highly-compact saprolite, which we took as originating from the Vijayan Complex bedrock gneiss^38,39^ and which marked the end of our excavation. Lithic artefacts were recovered in Units 10b-10 (n = 912), and our Kernel Density distribution model (Fig. 2E, Fig S1) suggests a concentration within these units. The majority of the artefacts recorded had their longest axes oriented horizontally or at low angles of dip (typically < 10° for artefacts > 7 cm) (see Fig. 2Aiv–v).

Units 9–4 represent the upper Latosol (red clayey sands) member of the IFm, separated from the lower gravel by a sharp, low-relief surface. Unit 9 extends for ca. 40 cm, consisting of coarse-grained, well-sorted sand with faint sub-horizontal laminations. Unit 8 overlies Unit 9 and is identical in terms of sediment type but has some notable mottling due to groundwater table fluctuation. Units 6–7 make up ca. 1.7 m of the latosol member and are composed of fine-grained compact sand with abundant pedogenic calcrete nodule inclusions. These two units are differentiated from each other by colour and their relative compactness, with Unit 7 being a deeper red and extremely compact due to Fe enrichment and CaCO_3_ cementation, respectively. Both units yielded stone artefacts at a density of around 300 lithics per cubic metre of sediment (Table 1). Units 5a-4, which range in thickness between 10–20 cm, make up the upper component of the IFm latosol layer. These units are less compact than the underlying Units 6 and 7, with more clay components and fewer calcrete inclusions. Unit 4 is notably less compact than the underlying Units 5/5a and yielded fewer artefacts at 77 lithic specimens per m^3^ sediment compared to 354. Above this is the shell layer (Unit 3), which extends between 20–30 cm across the excavation trenches. The shell, mostly bivalves (discussed in detail below), is imbricated at low angle (< 15°), and has a clayey sand matrix identical macroscopically to the overlying sediment of Unit 2. Units 1–2 are modern soils composed of well-sorted aeolian sand. Unit 2 has the densest artefact concentration, with an estimated density of 1105 stone artefacts per m^3^ of sediment (n = 610).

We obtained OSL dates for nine of the excavated archaeological units in Pathirajawela. Sampling and sample preparation were performed following standard procedures and further details are given in the Methods section and the SI. In addition, a portable OSL (POSL) luminescence profile was obtained to gain insights into the syn- and post-depositional processes involved in the site’s stratigraphical sequence. The samples from bottom to top of the sequence yielded ages between 24.8 ± 2.4 and 1.2 ± 0.1 ka ago (Fig. 2b; Table 1; Table S2). The dates are stratigraphically consistent except for two inversions. We obtained an age of 17.5 ± 1.0 ka ago for Unit 9 which is younger than the Unit 8 age of 19.4 ± 1.3 ka ago. In addition, Unit 3 has an OSL date of 8.9 ± 0.9 ka ago compared to 6.6 ± 0.4 ka ago and 7.6 ± 0.5 ka ago obtained for the underlying Units 5 and 6 respectively. Dose rate values for all samples except Unit 3 present similar values of ~ 2 Gy/ka. The presence of carbonates in Unit 3 could result in an underestimation of the annual dose rate (~ 1.1 Gy/ka, see Fig. S2), in turn overestimating the final age calculation. The previous C-14 date of 4.8–3.8 ka cal BP^14^ for this shell layer in Pathirajawela aligns well with our current chronostratigraphy.

The luminescence profile seen in Fig. 2C shows the POSL values for infrared stimulated luminescence (IRSL; red) and OSL (blue) measurements (methodological details are presented in SI). The OSL and IRSL POSL values show a good correlation increasing with depth, as expected in a sedimentary environment conforming to the law of superposition. The signal increases gradually with depth, indicating an ageing with depth trend. However, we note two important observations. Between Units 9 and 10, a major post-depositional change and reworking occurred, as suggested by the unexpected low POSL values, which correlates to the inverted date we observed for the unit. In addition, we observed that the luminescence signal did not vary from top to bottom of Unit 3 suggesting a relatively rapid deposition of this unit. We also conducted magnetic susceptibility measurements on the same samples. We observed a set of highs and lows in the value of magnetic susceptibility along the stratigraphic profile, notably three peaks in Units 2–3, Unit 7, and Unit 10 (Fig. 2D) The most parsimonious explanation is that the horizons of higher magnetic susceptibility correspond to intervals of higher concentration of magnetite, most likely due to increased production of pedogenic magnetite (or maghemite) in the soils^40–42^ during periods with more abundant moisture and vegetation.

### The Holocene shell midden

**Fig. 3:**
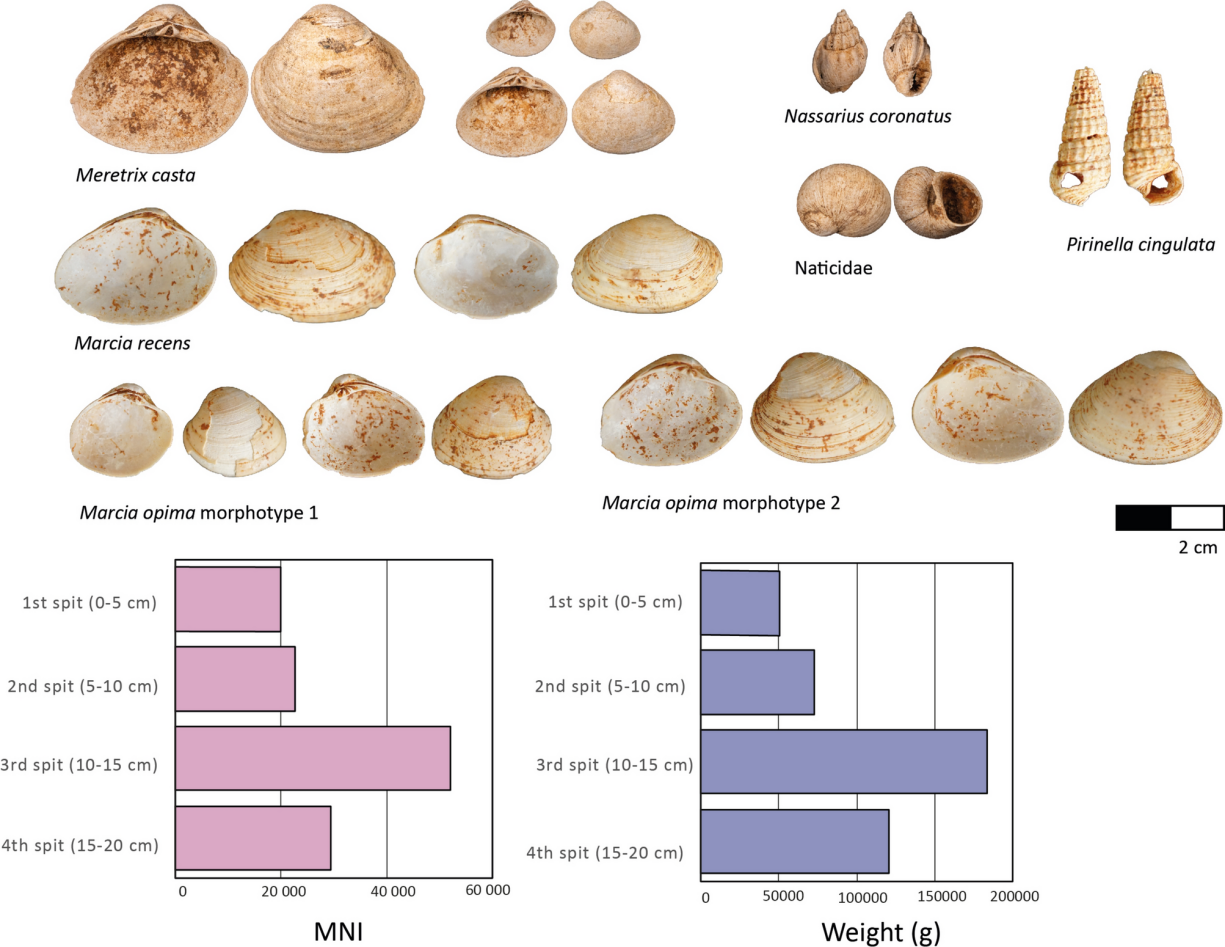
Different shell taxa identified from Unit 3 at Pathirajawela and the distribution of shell by MNI (left) and weight (rights) per 5 cm excavation spit (combined squares N5E1 and N5E2).

A total of 749,389.6 g (or 749.4 kg) of shell was recovered from the excavation of Unit 3 at Pathirajawela. From the two analysed squares, a total weight of 428,509 g and a total Minimum Number of Individuals (MNI) of 124,137 were recorded (Table 2). The vertical trends in MNI and weight data are very similar when these data are combined and assessed by 5 cm excavation spits (Fig. 3, see also Methods). The quantity of shell peaks in the third spit (10–15 cm below unit surface), followed by a decrease in the upper 10 cm, although the extent of this reduction in shell appears greater when measured by weight.

**Table 2:** Taxonomic distribution by MNI (left) and weight (right) of shells per 5 cm excavation spits in Unit 3 (combined squares N5E1 and N5E2).

Class	Family	Taxon	1st spit (0–5 cm)		2nd spit (5–10 cm)		3rd spit (10–15 cm)		4th spit (15–20 cm)		Total	
			MNI	Wt (g)	MNI	Wt (g)	MNI	Wt (g)	MNI	Wt (g)	MNI	Wt (g)
Bivalvia	Cardiidae	Cardiidae sp.					1	0.1			1	0.1
	Veneridae	*Meretrix casta*	18213	42668.7	21196	61897.3	47707	151226.4	27486	103421.5	114602	359213.9
		*Veneridae* spp*.*	1812	9343.65	1280	10447.06	3970	31173.66	1616	16392.78	8678	67357.15
		*Marcia recens*	7	9.9	45	101.7	120	326.92	169	535.74	341	974.26
		*Marcia opima* (morphotype 2)	1	2.9	15	62.4	45	222.5	53	262.28	114	550.08
		*Marcia opima* (morphotype 1)	1	2.6	9	46.6	18	112.9	19	120.99	47	283.09
Gastropoda	Acavidae	c.f. Acavidae	1	1.3							1	1.3
	Ariophantidae	c.f. *Cryptozona* sp.					1	0.4			1	0.4
	Cerithiidae	c.f. Cerithiidae			1	0.6					1	0.6
	Cyclophoridae	c.f. *Leptopomoides* sp.	1	0.1							1	0.1
	Nassariidae	*Nassarius coronatus*	24	3.0	17	3.36	27	11.39	5	2.51	73	20.26
	Naticidae	*Polinices* sp.			1	0.4					1	0.4
	Potamididae	*Pirenella cingulata*	61	19.1	30	16.88	145	58.01	38	13.34	274	107.33
	Turbinidae	c.f. Turbinidae					1	0.2			1	0.2
		Indeterminate gastropod	1	0							1	0
Totals			20122	52051.25	22594	72576.3	52035	183132.48	29386	120749.14	124137	428509

The assemblage exhibits a very low degree of taxonomic richness (Table 1; Fig. 3), with only 14 taxonomic categories recorded. It includes three terrestrial gastropods (MNI = 3, 2.2 g) and these likely represent natural incorporation into the deposit. The presence of a specimen morphologically consistent with Acavidae is interesting, as these terrestrial gastropods do not occur naturally in lowland arid/semi-arid areas of Sri Lanka. This is somewhat consistent with data from other shell deposits in the area, with *Acavus* being reported from Mini-athaliya^26,28^ and Kalametiya^43^. Three marine gastropods identified as Cerithiidae, Naticidae and Turbinidae, and one marine bivalve identified as Cardiidae, are similarly minimally represented (MNI = 4, 1.3 g). Of the remaining taxa, two medium-sized marine gastropods are comparatively well represented, these being *Pirenella cingulata* (MNI = 274, 107 g) and *Nassarius coronatus* (MNI = 73, 20.3 g). *P. cingulata* commonly reaches 3.5 cm in shell length and is often abundant on mudflats near mangroves and brackish waters, whereas *N. coronatus* inhabit sandy/silty substrates of intertidal to shallow subtidal zones and commonly reach 2.5 cm in shell length^44,45^.

The dominant taxon in Unit 3 is the brackish water bivalve *Meretrix casta* (114,602 MNI, 359,213.9 g), followed by specimens attributed to the family Veneridae (8678 MNI, 67,357.15 g). For the Veneridae specimens, three taxa with similar morphology have been identified as *Marcia recens* (341 MNI, 974.26 g), *Marcia opima* (morphotype 1) (47 MNI, 283.09 g) and *Marcia opima* (morphotype 2) (114 MNI, 550.08 g). *Marcia recens* inhabits sand and muddy-sand bottoms in the intertidal and sublittoral to a depth of 50 m^44^. Both morphotypes of *Marcia opima* are sub-trigonally ovate, however, morphotype 1 is slightly more ovate in outline. *Marcia opima* inhabits soft bottoms in intertidal and shallow subtidal waters, especially in protected coastal areas and near estuaries^44^. Considering the 5 cm excavation spits, *M. casta* has an MNI value that ranges from 90.18 to 93.81%, while the MNI for the combined Veneridae is between 5.5–9.01%, *P. cingulata* 0.13–0.3%, and *N. coronatus* 0.02–0.12%. This type of virtually mono-specific shell deposit occurs across the southern coast of Sri Lanka^25,28^.

Taphonomically, the shell across all excavation squares exhibits minor weathering and dissolution (see Fig. 3 for representative images per taxon), with no obvious evidence for natural predation (e.g., drill holes, repetitive breakage patterns). Less than 0.1% of the total assemblage across all taxonomic categories exhibited evidence for mechanical modification consistent with water rolling and natural transportation. Fragmentation patterns for the bivalves across the entire assemblage conform morphologically to breakage produced by post-mortem compression or compaction that can be attributed to diagenesis and accumulation of the overlying sediments. These factors are indicative of post-depositional and in situ degradation as opposed to processes those that would occur during natural deposition. The restricted number of taxa, and the paucity of small non-economic taxa, pumice, rounded gravels and/or corals^46,47^ also provide support for an interpretation of anthropogenic origin. Additionally, the proportion of articulated valves (as a contribution to MNI), highlighted by Harmsen^25^ and Weerarathne et al.^37^ as a defining feature in support of natural deposition, is particularly low as articulated valves contribute between 0.07 and 1.88% of the shell assemblage. Finally, the characteristics of the shell deposit itself do not conform to natural deposition, being clearly delineated with sharp stratigraphic boundaries, moderately densely packed, and with the shell showing no preferred orientation nor consistent internal bedding (Fig. 2A)^48^.

Taken together, this body of evidence, including the condition of the shell and lack of evidence for water-rolling or high-energy transportation, the shell deposit and assemblage characteristics, and the restricted taxonomic composition dominated by a single brackish water/estuarine species, points to an anthropogenic origin for the assemblage. The findings are not indicative of natural deposition of the shell material at Pathirajawela by the kind of large-scale, violent storm event required for transportation to a raised position on the coast located at c. 20 m ASL.

### Lithic assemblages

The excavation yielded a total of 4867 flaked stone artefacts across the thirteen archaeological units, with three distinct peaks in artefact concentration through the site sequence. Our kernel density estimation of the vertical distribution of lithic materials (Fig. 2E; Table 1) points to three distinct horizons of artefact concentration. The uppermost cluster spans Units 1–3 (dated to the last 5000 years) with a total of 1758 lithics recorded (619 artefacts/m^3^), followed by a middle concentration near the base of Unit 7, ca. 14.2 ka ago, (total for unit: 304 artefacts/m^3^; base only: 507 artefacts/m^3^) and finally a concentration that spans Units 10-10b dated to ca. 25 ka ago, within the sedimentary unit directly overlying the basal gravel layer (371 artefacts/m^3^). Interestingly, these correspond to the same units where we observed high magnetic susceptibility measurements which we hypothesize to correlate to periods of higher moisture or vegetation. Table 1 summarizes the total number of lithic artefacts recovered as well as artefact density per excavation unit.

**Fig. 4:**
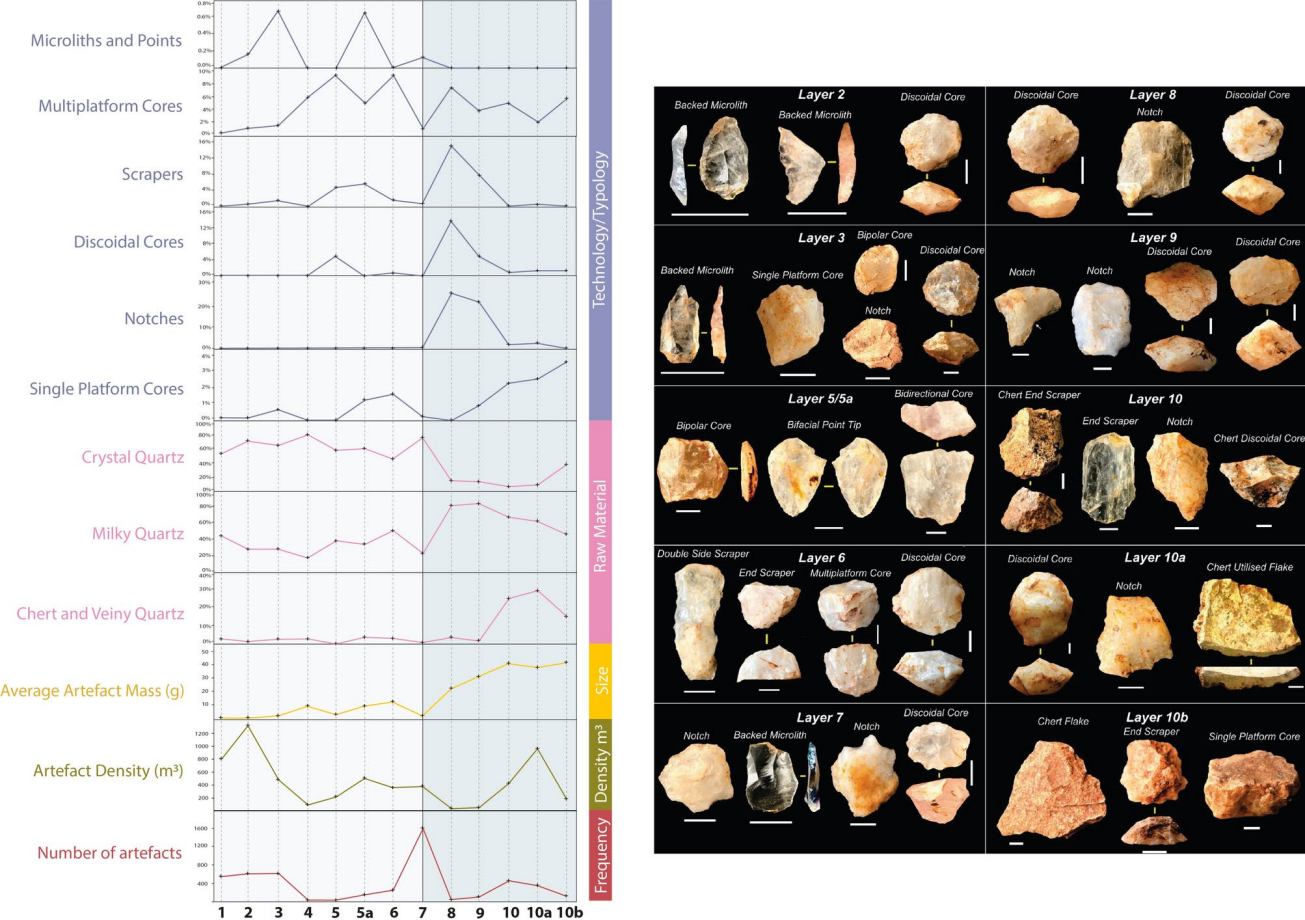
Overview of key changes in lithic materials through time at Pathirajawela and characteristic artefact types from each excavation units except Units 1 and 4 which yielded very few artefacts.

The lithic artefacts in Pathirajawela were manufactured from a range of raw materials, with clear (crystal) quartz dominant at 55% of the total assemblage. Milky quartz is the next most common stone type at 37.5%, while veiny quartz and chert are rare raw materials making up 6% and 0.3% respectively. Raw material proportions change appreciably over time (Fig. 4, S5–S6; Table S6), with clear quartz making up the bulk of the upper assemblage in Units 1–7, while milky quartz is dominant in the lower assemblage in Units 8-10a. Veiny quartz and chert also become common in these lower units, (from < 4% above Unit 8 to ~ 25% in lower units). Chert is practically absent in the upper units but rises to 3% in Unit 10a.

The Pathirajawela assemblage consists largely of flaked pieces (48%) (Fig. S7; Table S7), defined as artefact fragments derived from conchoidal fracture that cannot be oriented or identified to artefact type. High proportions of flaked pieces are expected in quartz assemblages as quartz tends to fragment upon impact to a greater degree than other raw materials. Broken and complete flakes are the next most numerous types (28% and 14%), followed by cores and fragments, retouched flakes, bipolar pieces (cores and flakes), redirecting flakes struck from rotated cores, and hammerstones and fragments. Two possible grindstone fragments were also found in Unit 3. The assemblage shows changes in assemblage composition that mirror those of South Asia more generally^49,50^, despite being manufactured predominantly on quartz. The lowest units, dated to ca. 24.8–19.4 ka, (8-10b) contain high proportions of scrapers, notches and dual hemisphere cores that fall somewhere between discoidal and semi-discoidal, alongside numerous single and multiplatform cores. Artefacts in the lowest units are large to medium-sized and show evidence of production from fist-sized or larger quartz cobbles. Chert artefacts are consistent with production from similar types of cores to those of quartz. The upper assemblage from Units 7–3 (from 14.2 ka) is microlithic in nature, containing mostly small-sized flakes and cores with rare backed microliths made from quartz. Units 1 and 2 (ca. 2.1–1.2 ka) are also small-sized and consistent with a microlithic assemblage, but backed microliths are absent, perhaps due to a smaller sample size. Discoidal cores also occur in the upper units and no blade cores are present. The backed microliths are clearly made from small flakes and not blades, consistent with a general absence of blade technology in Sri Lanka^14,21,51^. The overall diminution in the size (average mass) of artefacts through time is evident (Figs S8–S9).

Analysis of the complete flakes from the assemblage shows a number of changes in flake size, shape and technology over time, most notably decreasing size and increasing elongation, with flakes in the upper assemblage on average one-sixth the size of those in the lower assemblage and around 12% more elongate (Fig. S9–S10; Table S8–S12). Flake platforms show changes in type and preparation over time. Dihedral platforms are more common in the lower half of the site, particularly in Units 8-10b. This distinctive platform type is commonly associated with discoidal core manufacture due to the striking of ridges on the platform hemisphere of cores to produce short—often converging—flakes on the opposite hemisphere that run to the central apex of the core face. Dorsal scar orientations, indicative of flaking patterns and core reduction, also change through time. Weakly centripetal and bidirectional flake scar patterns (i.e. scars that originate from several points around the flake or core circumference but are too few in number (< 4) to determine a strong centripetal pattern^52^) become more common at the base of the site through Units 8–10b.

In terms of cores, multiplatform cores are the most numerous, representing 45% of the total core assemblage followed by core fragments, usually multiplatform fragments (Fig. S11–S14; Table S13–S16). Semi-discoidal and single-platform cores are the largest on average in the core assemblage, and multiplatform and discoidal cores are smaller on average, while bidirectional and bipolar cores are the smallest (Table S13). This pattern of diminishing core mass according to reduction technology is consistent with core reduction patterns worldwide^53–55^, where single platform cores are rotated after a time and become multiplatform cores. Much like the mass of complete flakes, the average mass of cores diminishes markedly through time (Fig. S12–S13), from 140-267 g in Units 10-10b, down to a mass of less than 32 g in the upper Units 1–7 assemblage. Like the flakes, there are also pronounced changes in the proportions of core types through time. Single platform cores are the most common in Unit 10B, with these decreasing as discoidal cores rise to prominence in Units 8 and 9. Multiplatform cores then dominate from Units 7–10, with a brief reappearance of discoidal cores in Units 5 and 2. Bipolar cores occur sporadically in Units 3, 5a, 7 and 9 but are always rare (Fig. S14; Table S13–S15). A comparison of core summary statistics between the upper (Units 1–7) and lower units (Units 8-10b) indicates cores are significantly larger in all dimensions in the lower units (Table S16). The amount of cortex on cores, the number of core rotations, the number of step or hinge terminations, parallel scars, shape or final platform angle do not differ significantly (see Table S9). This indicates that the main difference between cores in the lower units and the upper Microlithic units reflects a diminution in size over time.

Retouched flakes are among the largest flakes found at Pathirajawela, with a wide range of types usually made on milky quartz (50%) and clear quartz (34%), with the most common being notches (25.5%) and end scrapers (13.2%) (Tables S17). We observed clear changes in retouched flake typology in the site through time (Fig. S15). Notches dominate the lowest units, up 50–100% of the retouched assemblage in Units 8-10b. The proportion of notched artefacts gradually declines over time, and notches are largely absent above Unit 6. By contrast, scrapers rise in proportion as notches decline, peaking at 100% of the retouched assemblage in Units 5a and 1. Scrapers are entirely absent from Unit 4, but this is likely sample size-related since there are only 34 artefacts in this unit. Backed microliths in the form of asymmetric (pointed forms) and symmetric (lunate) forms are present in Units 2, 3 and 7. A single tip of a clear quartz bifacial point was also found in Unit 5a. Summary statistics for retouched flakes suggest substantial differences in size between retouched flakes in the upper and lower assemblages, but not in the shape of artefacts or in the nature or intensity of the retouch characteristics themselves (Table S18–S19). As above, this indicates that the assemblage contains essentially similar kinds of retouch throughout, the main difference being a dramatic reduction in size and changes in raw material through time, as well as the addition of backed microliths and bifacial point manufacture in the upper Microlithic assemblage above Unit 8, and more pronounced use of discoidal cores and single platform cores in the lower units below Unit 7.

## Discussion

Our investigation of Pathirajawela has produced the earliest well-dated record of human occupation of an open-air coastal site so far in Sri Lanka, with evidence of human activities from 24.8–1.2 ka. The discrepancy between the original thermoluminescence dates obtained by Singhvi et al.^36^ and the OSL ages we report here requires comment. As noted above, Singhvi and colleagues report ages of ca. 28.5 ka for the lateritised sand (at 1.8 m from surface) and ca. 64.4–74.2 ka date for the basal gravels (at 4 m from surface). Our OSL ages for equivalent stratigraphic units at comparable depths are 14.2 ± 1.1 ka and 24.8 ± 1.66 ka respectively. We suggest that the OSL ages are likely to be more robust in this region for several reasons. Firstly, it has been noted that there is a risk of producing TL age overestimates from certain sediments and depositional contexts due to incomplete bleaching (or incomplete optical resetting) of the samples at the time of deposition^56,57^. Further to this point, clay-dominated sediments, like those found at Pathirajawela, are more prone to aggregation. As the grains within the inner part of the sediment aggregate may be shielded from exposure to daylight, there may be insufficient zeroing of the luminescence signal. Similar erroneous results may also be obtained due to mineral (iron, manganese or carbonate) coating preventing daylight penetration into the quartz grains^58^. Although accounted for to a degree in the discussion of their methods, iron staining was a significant factor noted by Singhvi and colleagues^36^ for their Pathirajawela samples, suggesting that iron coating may have led to significant inaccuracy of the initial thermoluminescence dates. Additionally, other issues have affected the quality of TL dates produced on dune sand between 1985 and 1990 globally, including difficulties with dose-rate determination and reproducibility of the TL signals in quartz^59^.

Our OSL ages also broadly correlate with the chronology of the similar Teri sand deposits of Tamil Nadu in southeast India^5^. In this region there appear to be several phases of red dune deposition, an initial phase that falls prior to the LGM based on terrestrial snail shell radiocarbon dates (31–29 ka, 26–24 ka), followed by a large series of OSL ages that indicate formation during the Late Pleistocene (16–9 ka) and Mid to Late Holocene^60,61^. More recent work in Wilpattu National Park in northwestern Sri Lanka has suggested that the Basal Ferruginous Gravels (forming c. 19–15 ka) identified in that area cannot be correlated with the basal gravel deposit below red dune sands in the Bundala region since the latter pre-dates the LGM^5^. Our OSL ages for the lateritised (red) sand and basal gravels at Pathirajawela fall more in line with the ages obtained from southern India and provide a much stronger correlation with northwestern Sri Lanka.

The OSL ages that bracket the shell deposit are 2.1 ± 0.2 ka (above) and 6.6 ± 0.4 ka (below), with an OSL age from the middle of the shell deposit itself at 8.9 ± 0.9 ka. Our OSL age for the shell deposit is therefore viewed as being anomalous, particularly given that Deraniyagala^21^ produced a radiocarbon date for the Pathirajawela shell deposit of 4500 ± 170 BP (PRL-107), which calibrates to 4.8–3.8 ka (2σ) using the Marine20 calibration curve^62^ with a Delta R correction value of 133 ± 65^25^. As noted above, the shell deposit OSL age is likely an overestimate due to the presence of carbonates in Unit 3 that result in an underestimate of the annual dose rate.

The original radiocarbon age presented by Deraniyagala^14^ for Pathirajawela conforms to other dates obtained from shell deposits on the southern coast of Sri Lanka. Radiocarbon ages produced on shell deposits in the region surrounding Pathirajawela largely fall between 1.9 ka and 6.2 ka cal BP^21,25,26,43,63^, which effectively correlates with the OSL ages for the stratigraphic units immediately above and below the shell deposit from our excavation. This chronology also reflects processes of postglacial sea level rise and subsequent lagoon/estuarine development on the southern Sri Lankan coast. Three mid-Holocene highstands (c. 6.2–5.1 ka; c. 4.4–3.9 ka; c. 3.3–2.3 ka) were identified by Katupotha^3^, with relative sea level peaking at c. 2 m ASL following rapid sea level rise from c. 17 ka into the early Holocene. This pattern is broadly supported by modelled eustatic sea level for Sri Lanka^64^, which indicates a c. 2.5–3 m increase that ceased around 4 ka, followed by a marine transgression. This trend for southern Sri Lanka was also replicated in modelled data for southeast India and the Maldives^64^.

The Pathirajawela shell deposit was initially identified by Deraniyagala^21^ as a cultural midden, however, the anthropogenic origin of this site and many others across the southern coast of Sri Lanka have been called into question^4,25^. Although natural storm deposits and cheniers undoubtedly occur across this dynamic coastline, these interpretations have not been based on detailed, systematic shell analyses. Our findings, drawing on taxonomic and taphonomic observations, suggest an anthropogenic rather than natural origin for the Pathirajawela deposit. Given the parallels between the Pathirajawela shell assemblage and others along the southern coast, our findings also highlight the probable anthropogenic origin of many of Sri Lanka’s disputed shell middens, though further similarly detailed study of additional middens is required to confirm this. Both Deraniyagala^21^ and Harmsen^25^ note that there is no clear evidence for size-selective harvesting in the shell deposits situated along the southeast coast of Sri Lanka. While not yet quantified for Pathirajawela (as this is the subject of an ongoing in-depth study), our observations of the shell assemblage support that suggestion. Given the volume and density of the shell material, combined with a single taxon-dominated deposit in the order of c.95%, the data point to a mass harvesting strategy in place to take advantage of a potentially biomass-dominant resource^65^. Our findings suggest that people inhabiting this area were able to adapt their economic systems to suit shifting coastal ecosystems and actively exploit changing near-shore and estuarine resources during the Holocene marine transgression.

The ca. 25 ka Pathirajawela lithic sequence stands in contrast to the sequences documented from the cave sites in the Wet Zone region in that it is marked by a series of successive changes in key techno-typological forms and raw materials, with an important technological change in Unit 7 demarcating the appearance of microlithic artefacts at the site. The Pathirajawela sequence shows a progressive decline in mean artefact mass, with the most pronounced decrease taking place between Units 8 and 7 following the appearance of microliths. The sequence also exhibits a distinct change in core technology with exclusive flaking of single platform cores in the lower units, through discoidal cores and then the predominance of multiplatform cores in younger units. This is in contrast to the microlith-dominated sequences identified at rainforest sites such as Fa Hien-lena^51^ and Kitulgala Beli-lena^14^ which continued almost unchanged from ca. 48 to 5 ka cal BP and is hypothesised to reflect composite projectile technologies that facilitated specialised hunting of arboreal/semi-arboreal prey. To our knowledge, the Pathirajawela sequence is the first dated lithic sequence, spanning the LGM to mid-Holocene, exhibiting distinct techno-typological changes anywhere in Sri Lanka. It also appears to represent the only assemblage spanning this period from coastal or near-coastal environments across South Asia more broadly. As such, more research is needed to determine what brought about these changes, requiring a larger number of assemblages from well dated and stratified coastal sites with occupation dating from the late Pleistocene. That said, our results hint at a possible link between the major shift in lithic technology and adaptive processes connected to ecosystem changes following the end of the LGM. Unlike the Fa Hien-lena and Kitulgala Beli-lena sequences, which are associated with relative environmental and resource stability through time, the Pathirajawela sequence occurs in the context of substantial environmental and economic change in a contrasting coastal landscape. We hypothesize that the increasing environmental complexity and restructuring to a more mosaic type of habitat distribution on and near shore following the LGM may have been one of the drivers of changes in lithic technology.

**Fig. 5:**
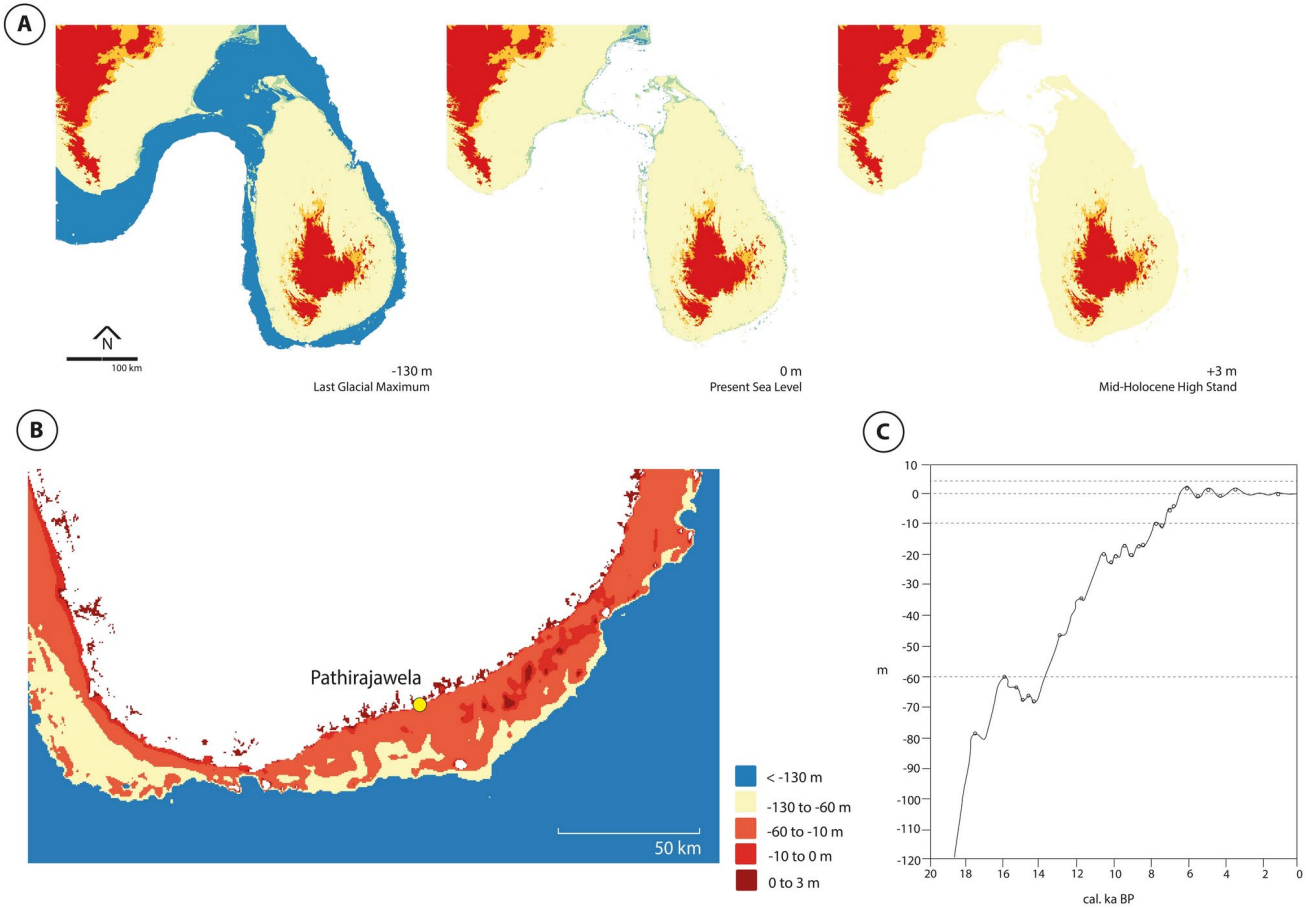
Changes of land extent in Sri Lanka (**A**), particularly of the island’s southern coast (**B**) following sea level changes modelled using bathymetry data from GEBCO_2023 Grid^58^. We used sea level oscillation data from Katupotha^3,4^ (**C**), redrawn from ref^4^).

Overall, our investigations have produced the earliest reliably dated and archaeologically studied coastal site in Sri Lanka. Before and during the Last Glacial Maximum, bathymetric data^66^ and provisional sea level models^3,4^ suggest that Pathirajawela was located ca. 22 km away from the coast (Fig. 5). By 16 ka BP when the sea level is estimated to have been 60 m below the current level, the site would have been located just ca. 12 km away from the sea. Rapid sea level rise commencing from ca. 14 ka BP resulted in the continental shelf becoming increasingly submerged, culminating in the development of a mosaic of intertidal habitats and the subsequent formation of estuarine environments. This coincides with a major change in lithic technology at the site, specifically a predominance of microliths. From ca. 4.8 ka BP, coinciding with the peak of the Holocene marine transgression, human activities at Pathirajawela were largely geared—although likely not exclusively—towards estuarine ecosystems, particularly the exploitation of molluscan resources. This site is located effectively at an ecotonal boundary, one that would have facilitated access to a variety of terrestrial, freshwater and marine ecosystems. As has recently also been noted^31^, the Holocene economic structure on the Sri Lankan south coast would have been more complex than the shell deposits alone suggest. While the evidence does point towards an intensification in estuarine resource use as a part of human adaptation to changing nearshore environments with sea level rise, foraging in estuarine and marine habitats would likely have formed just one part of a broader, more elaborate economic system.

Our findings highlight the value of looking beyond Sri Lanka’s southern interior to examine long-term human habitation of the island. We extend the reliable record of human habitation of the coast to 25 ka, demonstrating that intensified coastal resource exploitation occurred in the context of rising sea levels as Sri Lanka re-emerged as an island after the LGM. Future research should accordingly focus on systematic investigation and dating of more coastal sites not just in the island’s southern region, but also the western, eastern and northern coasts where very limited research has so far been undertaken (see Fig. 1B), as well as comparative studies looking at sites in southern India. Current anthropogenic climate change and global sea level rise make much of this research more urgent, not only in terms of threatened cultural heritage in coastal regions, but also in terms of understanding the long-term impact of sea level rise on human communities globally.

## Methods

### Excavation

Six 1 × 1 m trenches (N3E1, N3E2, N4E1, N4E2, N5E1, N5E2) were opened during the 2019 Pathirajawela field season following the grid system established during the 1970s excavations. Excavations were conducted using hand tools and removing and recording individual sediment contexts. Where the depth of deposit of a single unit was extensive, the unit was subdivided into arbitrary spits to increase the resolution of artefact and sediment sampling, for instance, the shell midden layer (Unit 3) was subdivided into four 5 cm spits. Artefacts recovered during the course of the excavation were bagged by category, or individually for special finds, and labelled appropriately. Three-dimensional recording of the sediment deposits and major inclusions was done through the use of a Leica TS16 Total Station and a CS20 controller, with absolute 3D coordinates recorded for each artefact as well as individual context, or spit within a context. Digital context recording was simultaneously carried out at the site using an existing Android platform and a form developed for the MPI-GEA. This recorded: sediment compaction, sediment moisture, sediment colour intensity, hue and colour, major and minor sediment composition, presence of major non-cultural inclusions, post-depositional alterations, sediment bedding, and artefact presence. Sediment density was calculated from total station measurements. All artefacts recovered were assigned individual accession numbers and barcodes and information about their provenance were recorded in a digital database.

### Shell midden analysis

Before analyses, all samples were washed to remove excess sediment and air-dried for 24–48 h. Taxonomic attributions were made following the descriptions and/or illustrations provided in published keys^69–73^. Specimens were attributed to taxonomic categories (e.g., to species, genus, or family) based on the preservation of identifiable diagnostic features. These attributions were checked using the World Register of Marine Species^74^ to ensure that the most up-to-date nomenclature was recorded. A preliminary taphonomic assessment of the shell was undertaken, including edge damage/water rolling, burning, damage by marine sponges, polychaete worms, drilling and boring, presence of vermetids (sessile gastropods which may attach to other shells), hermitting by crabs, root etching, and dissolution of the shell (chalkiness). Although not quantified at this stage, this was done to determine the likely origin of the material (natural vs cultural deposition) and the taphonomic processes impacting the assemblage. Two quantitative measures, Minimum Number of Individuals (MNI) and weight (g), were recorded for each taxonomic category from squares N5E1 and N5E2. The umbo was used as the non-repetitive element for all bivalve taxa, with the spire/apex, aperture and columella used for the gastropod MNI calculations. Total shell weights were also recorded for squares N3E1 and N4E1. Due to the large volume of material recovered from the Pathirajawela shell midden excavation we focus here on the data from squares N5E1 and N5E2. These two squares were also excavated in four arbitrary 5 cm units, facilitating a degree of vertical assessment. Further work will be undertaken on the Pathirajawela assemblage, including a full quantified taphonomic assessment, recording the Number of Identifiable Specimens (NISP) for each taxon, and biometric (size/shape) analyses.

### Lithic analysis

The analysis was undertaken in two stages. The first stage involved the identification, counting and weighing of all artefacts by bag and context using common types and classes found in South Asia. This data was entered into a Microsoft Excel spreadsheet by bag and context. The second stage involved the measurement and recording of all complete artefacts > 2 cm using the Lotus Approach database and protocols published in Clarkson^75^. Data were then analysed in Excel and SPSS. Important artefacts and characteristics of the assemblage were photographed and compiled in CorelDraw and CorelPaint and illustrated as line drawings in Procreate on an iPad Pro.

### Spatial analysis

Kernel density analysis is a widely used method in GIS for estimating the density of features (e.g., point-based data) in the neighbourhood of those features. It applies a kernel function on each observation point and calculates the estimated values around each output grid cell within a kernel window. The highest value is at the location of the point, and it decreases with increasing distance from the point, reaching zero when the search radius (bandwidth) from the observation point is exceeded.

The objective of applying this spatial analysis in Pathirajawela was to detect hot spots and breaks in the distribution of the recovered lithic materials, as well as to determine the dispersion patterns of the lithic features within the excavated area. The analysis was completed using the Kernel Density tool in ArcMap 10.5.1 @ArcGIS. The spatial calculations were reduced to only 3 excavation grids (N3E1, N4E1 and N5E1) covering a total area of 3 × 1 m and reaching a depth of 4.15 m. Only finds plotted with the total station were used for the calculations, while specimens recovered through flotation were excluded from the analysis.

Given the two-dimensional approach of Kernel density, two models of calculations were applied: (i) vertical density model, and (ii) horizontal density model. The first model (i) refers to the vertical distribution of features in the xy-plot. This model is well suited to evaluate the distribution of objects within individual depositional layers due to their horizontal orientation in the site. The choice of the optimal bandwidth has a great impact on the results which may avoid an under-smoothed and noisy surface or, on the contrary, an over-smoothed and generalised surface. However, there is no golden rule for estimating an optimal bandwidth. The spatial variant of Silverman’s Rule of Thumb used for Kernel Density estimation in ArcMap provided too general and over-smoothed results for our research area. Therefore, a series of models with different bandwidths in the range between 8 and 30 cm were tested. Finally, the fixed bandwidth of 20 cm with a cell size of 16 cm^2^ was chosen, which best reflects the density estimates for the excavated area. For display purposes, the output was interpolated using Bilinear Interpolation for continuous data.

A horizontal density model (ii) was generated to evaluate the results of the vertical section and to estimate the possible hot spots and distribution of features at the horizontal level within individual archaeological units. Given the different volumes and number of finds in each unit, the analysis was undertaken according to fixed-defined artificial strata in equal-sized 41 cm packages. In this way, the excavated sequence could be divided into 10 packages of equal size and thickness. The same fixed settings with a bandwidth of 20 cm were used for the calculation as for the vertical density model. However, the statistical values were less comparable between both models given the different sizes of the kernel areas. The Nearest Neighbour interpolation was used to visualise the hotspot estimates to minimise the distortion at the edges of the kernel window. Though the estimated surface still provides a slightly under-smoothed and over-clustered surface due to the relatively narrow bandwidth interval with the area and number of features, it best reflects the distribution when comparing all packages.

### Luminescence dating

#### Sampling and sample preparation

A total of nine samples were collected for OSL dating. They were processed and analysed at CENIEH Luminescence Laboratory (Burgos, Spain). Samples for Equivalent Dose (D_e_) analyses were taken using a light-proof 20 cm long metal or PVC tube with a diameter of 7 cm, which was hammered into the trench’s outcrop. A bulk sediment sub-sample for external dose rate calculation was also collected from each sampling point for high-resolution gamma spectrometry (HRGS) analysis. The remaining hole in the sediment was used for *in-situ* gamma spectrometry measurements. Sample preparation was performed following standard procedures in a dark lab under subdued red-light conditions to avoid luminescence signal depletion before OSL measurements. About 2 cm of the sediment that might have been exposed to daylight during the sampling process were removed on each side of the sample tubes. The sample preparation protocol consists of (1) sieving to separate the target grain size, (2) chemical treatment with HCl (32%) to remove carbonates and with H2O2 (35%) to eliminate organic material, (3) high-density liquid separation (2.70 g/cm^3^, 2.62 g/cm^3^, 2.58 g/cm^3^) to remove heavy minerals and to separate quartz from feldspars, (4) hydrofluoric acid treatment with 40% for 40 min, and (5) final dry re-sieving. Quartz grains were mounted on stainless steel discs and fixed using a 2 mm patch of silicone oil. A separate bulk sediment sub-sample was used for water content analysis, and subsequently, this sample was ground for gamma spectrometry measurements.

#### Equivalent dose determination

OSL dose evaluation on quartz coarse grains (90–125 µm) was carried out using the Single Aliquot Regenerative-dose (SAR) protocol^76^. Measurements were made using blue LEDs (470 nm) for stimulation and a Hoya U-340 filter for detection. Before D_e_ measurements, a pre-heat plateau test was performed with increasing temperatures between 180 and 260 °C (held for 10 s) and a cut-heat 20 °C below the preheat temperature. A dose recovery test was conducted on every sample after 2 h of bleaching in a Hönle UVA cube 400 solar simulator. Aliquots were irradiated with a known beta dose, equal to the estimated D_e_. Feldspar contamination was tested using IR stimulation and no detectable signal was observed. All datasets with recycling ratios and test dose uncertainties within 10% were accepted for palaeodose calculations (Table S2). Equivalent doses were determined with Analyst software v4.31.9^77^ by fitting a sum of two exponential functions to the dose–response curves. A central age model (CAM^78^) was applied and a three-parametric minimum age model (MAM^78^) was applied to the samples with overdispersion (OD) values > 25%.

#### Dose rate and age calculation

The total environmental dose rate (Table S2) was determined by both *in-situ* field measurements and laboratory analysis. The environmental gamma dose rate was measured in the field using a portable in-situ gamma spectrometer (Canberra InSpector 1000 coupled with a 1.5*1.5-inch NaI (Tl) probe), calculated with the “threshold” technique^79^. Natural activities of 238U, 232Th and 40 K were measured for ~ 100 g of homogenised dry ground sediment using a Canberra High-Purity Germanium Gamma-Ray Spectrometer (HPGe). Beta measurements were performed with a Risø low-level beta multi-counter. The software DRAC v1.2^80^ was applied for dose rate and age calculation using the conversion factors of Guérin and colleagues^81^, and the alpha and beta attenuation factors employed by Brennan et al.^82^ and Guérin et al.^83^. Natural water contents were calculated relative to the dry mass^84,85^ and the etching attenuation factors of Brennan^82^ were applied. The cosmic dose rate contribution was assessed after Prescott and Hutton^86^. All ages are given with the 1σ uncertainty.

#### Magnetic susceptibility measurement

Low field, bulk magnetic susceptibility measurements were performed on a MFK1-FA system (AGICO) using powered samples contained in plastic vials, at the paleomagnetism laboratory of the CENIEH.

## Supplementary Information

Supplementary Information.

## Data Availability

Data Availability: All data generated or analysed during this study are included in this published article and in the supplementary information file.
