# Supplementary material for: Early Sri Lankan coastal site tracks technological change and estuarine resource exploitation over the last ca. 25,000 years
Source: Sci Rep. 2024 Nov 4;14:26693. doi: 10.1038/s41598-024-77504-5 (PMC11535000; doi:10.1038/s41598-024-77504-5)
Supplement: Supplementary file 1 — Supplementary Information. [file 41598_2024_77504_MOESM1_ESM.docx]

Supplementary Materials for

**Early Sri Lankan coastal site tracks technological change and estuarine resource exploitation over the last ca. 25,000 years**

*Noel Amano^1^, Patrick Faulkner^2^, Oshan Wedage^3^, Chris Clarkson^4^, Dambara Amila^3^, Miren del Val^5^, Dovydas Jurkenas^1^, Alexander Kapukotuwa^3^, Gloria I. López^5,6,7^, Josep Pares^5^, M. M. Pathmalal^8^, Tam Smith^4^, Martin Wright^2^, Patrick Roberts^1,9^, Michael Petraglia^10,4,11^, Nicole Boivin^1,4,12^*

*^1^Max Planck Institute of Geoanthropology, Jena, Germany*

*^2^School of Humanities, Faculty of Arts and Social Sciences, University of Sydney, Sydney, Australia.*

*^3^Department of History and Archaeology, University of Sri Jayewardenepura, Gangodawila, Nugegoda Sri Lanka*

*^4^School of Social Sciences, University of Queensland, Brisbane, Queensland, Australia*

*^5^Spanish National Research Center for Human Evolution (CENIEH), Burgos, Spain*

*^6^Colombian Geological Society, Colombia*

*^7^Nuclear Affairs Directorate, Colombian Geological Survey, Bogotá, Colombia*

*^8^Department of Zoology, University of Sri Jayewardenepura, Gangodawila, Nugegoda, Sri Lanka*

*^9^isoTROPIC Research Group, Max Planck Institute of Geoanthropology, Jena, Germany*

*^10^Australian Research Centre for Human Evolution, Griffith University, Brisbane, Queensland, Australia*

*^11^Human Origins Program, National Museum of Natural History, Smithsonian Institution, Washington DC, United States*

*^12^Griffith Sciences, Griffith University, Brisbane, Queensland, Australia*

Table S1. Radiometrically dated archaeological sites in Sri Lanka

| Site No. | Name | District, Province | Latitude | Longitude | Vegetation Zone | Dates (BP) | Reference |
| --- | --- | --- | --- | --- | --- | --- | --- |
| 1 | Fa Hien-lena | Kalutara, Western Province | 6.6016667 | 80.2183333 | Wet Zone | ca. 48,000 - ca. 38,5000; ca. 12,500-5400 | Wedage et al., 2019 |
| 2 | Batadomba-lena | Ratnapura, Sabaragamuwa | 6.7765065 | 80.3959493 | Wet Zone | ca. 39,000 to ca. 10,000 | Perera et al., 2011; Roberts et al., 2017b |
| 3 | Kitulgala Beli-lena | Kegalle, Sabaragamuwa | 7.0022371 | 80.4357814 | Wet Zone | ca. 44,900-31,600; ca. 17,100-8,000 | Wedage et al., 2020 |
| 4 | Pothgul-lena | Gampaha, Western Province | 7.12573 | 80.1958755 | Wet Zone | ca. 14,000-9,800 | Adikari, 2010 |
| 5 | Alu-lena | Attanagoda, Sabaragamuwa | 7.2429064 | 80.389469 | Wet Zone | ca. 10,300 | Wijeyapala, 1997; Deraniyagala, 1992/2007 |
| 6 | Alugal-ge | Ratnapura, Sabaragamuwa | 6.59 | 80.68 | Wet Zone | ca. 5400 | Somadeva et al. 2008 |
| 7 | Lunugal-ge | Ratnapura, Sabaragamuwa | 6.57 | 8.72 | Wet Zone | ca. 5900 | Somadeva et al. 2008 |
| 8 | Kuragala-lena | Balangoda, Sabaragamuwa | 6.6261998 | 80.8679029 | Intermediate Zone | ca. 15, 100- 5900 | Eregama, 2022; Roberts et al., 2015; Stock et al., 2022 |
| 9 | Udupiyan Galge | Ratnapura, Sabaragamuwa | 6.58 | 80.82 | Intermediate Zone | ca. 9,500 | Deraniyagala, 1992/2007 |
| 10 | Bellanbendi Palassa | Ratnapura, Sabaragamuwa | 6.5 | 80.81 | Intermediate Zone | ca. 12,300-11,200 | Derayanigala and Kennedy, 1972; Deraniyagala, 1992/2007 |
| 11 | Potana | Matale, Central Province | 7.96 | 80.74 | Dry Zone | ca.5900- ca.5700 | Adikari 1994a |
| 12 | Aligala | Matale, Central Province | 7.95 | 80.75 | Dry Zone | ca. 5500 | Adikari 1994b |
| 13 | Mini-athiliya | Hambantota, Southern Province | 6.1215722 | 80.9481778 | Dry Zone | ca. 5000-2800 | Harmsen, 2017; Kulatilake et al., 2014; 2018; Roberts et al., 2022 |
| 14 | Kalamatiya | Hambantota, Southern Province | 6.110375 | 80.9521333 | Dry Zone | ca. 5000-3900 | Harmsen, 2017 |
| 15 | Pallemalala | Hambantota, Southern Province | 6.2265886 | 81.2034643 | Dry Zone | ca. 4700-2500 | Harmsen, 2017; Kulatilake et al., 2018 |

***Spatial Analyses of Lithic Finds***

Our vertical Kernel density estimation model (i) identified three significant lithic artefact density patterns indicating varying types of artefact deposition and distribution. The areas in between show breaks or random and sparse distribution of lithic finds. The horizontal density model (ii) also confirms the observations obtained from the vertical model. The results of both models indicate the most distinct distribution of features described as density patterns in the following areas:

1. The lower density pattern is located at the bottom of the cultural unit and clearly corresponds to Units 10, 10A and 10B. Accordingly, these units can be considered as a single unit package and are characterized by a comparatively high density and a very regular distribution of lithics. Even though the surface appears more clustered in the horizontal density model, the continuous horizontal distribution of lithics in all directions is to be expected.
2. The middle-density pattern is located slightly above the base of Unit 7 and is distinguished by the largest number of the finds, most of which are concentrated in the lower part. The find distribution shows a clear find stratum of about 50 cm thickness but with some irregularity and minor hot spots within it. Like the lower density pattern, the continuous horizontal distribution of lithics in all directions is to be expected as well. This density pattern marks a clear break between Unit 7 and the lower sedimentation sequence. The highly clustered surface in the upper part of this unit suggests similar depositional processes as observed in the upper-density pattern.
3. The upper-density pattern spans Units 1, 2, and 3. Both the vertical and especially the horizontal models clearly show “pocket” formations and local accumulations that are presumably of a secondary nature. The horizontal model (ii) indicates the vertical stretch-out of these pockets. The same clustered patterns were also evident in the test models using wider bandwidth intervals and more smoothed surfaces. These three units have been exposed to the same processes, most likely due to material relocation in the vertical axis. From the density point of view, this density pattern should be considered as one package. Even the transition zone between the upper and middle-density patterns (in contrast to the middle/lower density patterns) is diffuse and contains multiple small local peaks and fin scatters, whose interpretation as lithics displacement in vertical and/or horizontal directions is possible.

***Luminescence Dating and portable OSL reading***

Dose recovery preheat-plateau tests were performed and the most appropriate preheat temperatures are specified in Table S2. Feldspar contamination was tested using IR stimulation and no detectable signal was observed. The OSL signal was dominated by the fast component. The number of aliquots passing the rejection criteria varied between 63% and 96%. Overdispersion (OD) values vary between 15% and 41%. Samples resulting in a high dispersion of the obtained D_e_ values distribution (OD < 25%) (Table S3) suggest incomplete bleaching of the sediment before deposition, and hence, the Minimum Age Model (MAM) was calculated. Equivalent dose (D_e_) estimates are presented in Table S3 together with environmental dose rates and final ages.

***Luminescence Profile***

The Portable OSL (POSL) Reader (Sanderson and Murphy, 2010) was developed to quickly assess in the field whether a sample had any OSL signal or not, prior to full time- and labor-intensive laboratory preparation and measurement of every single sample for full OSL-dating. This quick reader (no radiation source) only measures OSL and IRSL signals (photon counts) directly on large bulk samples that are emitted from quartz and feldspar minerals embedded in the untreated sample. Thus, signal intensity can be used as a proxy for relative age (Stang et al., 2010) (as the POSL does not measure De but rather photon counts) considering environmental dose rates and mineral sensitivity (which is dependent on numerous physical and chemical factors directly related to the sample provenance and erosion-transport-deposition processes). Also, depletion rates and the OSL/IRSL signals ratio can also be used to understand the depositional history of the sediments, i.e., patterns of natural and/or anthropogenic reworking, syn- and post-depositional changes within the stratigraphy, potential diagenesis and even mineralogical variations (Stone et al., 2015; Kinnaird et al., 2017; Munyikwa et al., 2012).

Sampling is relatively simple as it depends on the stratigraphy and the degree of resolution needed or wanted. All sampling is to be done under tight light conditions (black tarp). In our case, small 10 g samples were scraped out along trench face N4E1, after cleaning the first 2 cm of any sunlight exposed sediment. A total of 42 samples were collected in small black ZIP bags. Prior to measurement, at the CENIEH´s Luminescence Dating Laboratory, samples were homogenized and quickly oven-dried at 60°C. Each POSL measurement takes about 5 minutes from beginning to end.

42 samples for Portable OSL reader (POSL) of 15 – 20 g were collected at 0.1 m intervals along the entire profile. Samples were collected into black light-tight bags under a light-tight cover to avoid depletion of the luminescence signal. All polymineral samples were dried and gently crushed before mounting a monolayer sample on a Petri dish 5 cm in diameter ensuring a constant distance to the PM tube.

All samples were measured on a SUERC portable OSL reader in a laboratory under subdued red light, using the CW Proxy protocol described by Sanderson and Murphy (2010) (Table S3) which included a double depletion of the IRSL signal followed by a 2-step OSL measurement. This instrument comprises a light-tight sample chamber where samples can be stimulated with photons from arrays of light-emitting diodes in the blue (470 nm) and infrared (880 nm) portions of the electromagnetic spectrum (Muñoz-Salinas et al., 2011). A single photon counting photomultiplier, filtered using a 12 mm Schott UG 11 filter is used to register luminescence signals during pulsed stimulation (Sanderson and Murphy, 2010).

The variation of the luminescence signal intensities with depth is plotted to contextualise sediment stratigraphy and can be used to interpret sedimentary processes (Munyikwa et al., 2021).


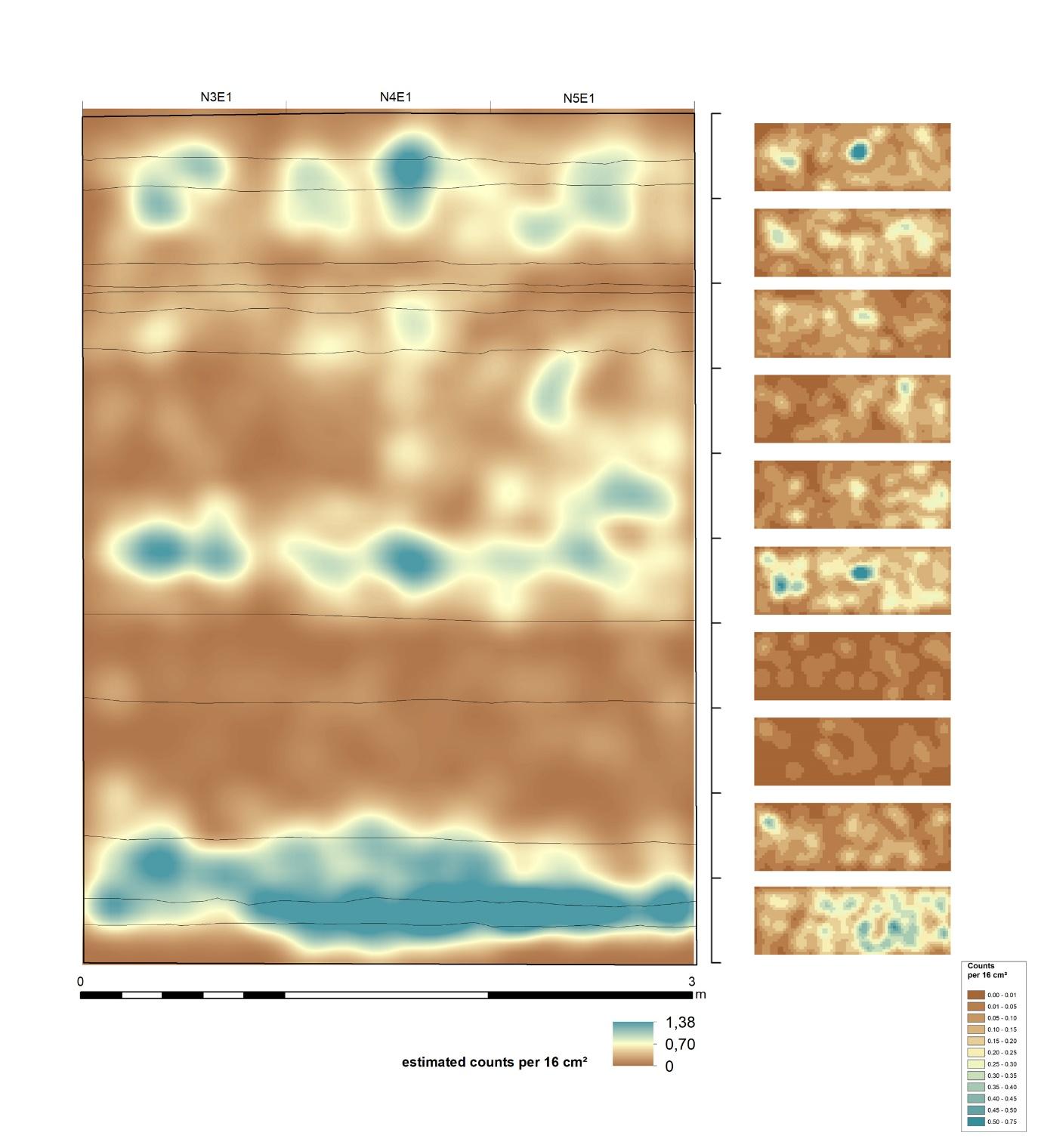


Figure S1. The vertical density model (i) on the left, and the horizontal density model (ii) on the right.

Table S2: Compilation of measurement data, including (i)Pre-Heat temperature, (ii) ^238^U, ^232^Th and ^40^K content measured by laboratory gamma spectrometry using a HPGe gamma spectrometer, (iii) calculated water content, (iv) Recycling ratio and Recuperation (%) and (v) Dose recovery test results

| Sample name | Lab.- Nr. | Pre-heat temperature (°C) | ^238^U  (ppm) | ^232^Th  (ppm) | ^40^K  (%) | | Water content (%) | Recycling ratio | Recuperation (%( | Dose recovery (%) |
| --- | --- | --- | --- | --- | --- | --- | --- | --- | --- | --- |
| BDP2019-N5E1-U1-OSL1905 | LM19128-05 | 240 | 2.75 ± 0.04 | 12.76 ± 0.31 | | 1.41± 0.04 | 8 ± 5 | 1.01 ± 0.04 | 0.71 ± 0.20 | 94.0 |
| BDP2019-N5E1-U2-OSL1907 | LM19128-07 | 220 | 2.75 ± 0.04 | 12.76 ± 0.31 | | 1.41± 0.04 | 9 ± 5 | 1.03 ± 0.04 | 0.49 ± 0.17 | 98.7 |
| BDP2019-N5E1-U3-OSL1902 | LM19128-02 | 220 | 0.81 ± 0.01 | 3.77 ± 0.06 | | 0.40 ± 0.01 | 5 ± 5 | 1.04 ± 0.04 | 0.50 ± 0.09 | 98.4 |
| BDP2019-N5E1-U5-OSL1906 | LM19128-06 | 220 | 2.89 ± 0.03 | 12.39 ± 0.28 | | 1.52 ± 0.04 | 7 ± 5 | 1.02 ± 0.03 | 0.61 ± 0.08 | 96.3 |
| BDP2019-N5E1-U6-OSL1903 | LM19128-03 | 220 | 2.75 ± 0.04 | 12.76 ± 0.31 | | 1.41± 0.04 | 6 ± 5 | 1.03 ± 0.03 | 0.27 ± 0.06 | 93.7 |
| BDP2019-N3E1-U7-OSL1904 | LM19128-04 | 220 | 2.90 ± 0.05 | 10.73 ± 0.28 | | 1.27 ± 0.04 | 5 ± 5 | 1.03 ± 0.03 | 0.38 ± 0.04 | 96.2 |
| BDP2019-N4E1-U8-OSL1908 | LM19128-08 | 220 | 3.27 ± 0.04 | 12.24 ± 0.31 | | 1.54 ± 0.04 | 8 ± 5 | 1.00 ± 0.03 | 0.23 ± 0.04 | 94.5 |
| BDP2019-N5E2-U9-OSL1901 | LM19128-01 | 240 | 2.51 ± 0.04 | 10.80 ± 0.28 | | 1.52 ± 0.04 | 8 ± 5 | 1.00 ± 0.03 | 0.30 ± 0.05 | 96.6 |
| BDP2019-N4E1-U10-OSL1909 | LM19128-09 | 260 | 2.17 ± 0.04 | 11.41 ± 0.30 | | 1.22 ± 0.04 | 6 ± 5 | 0.94 ± 0.03 | 1.14 ± 0.05 | 102.0 |

Table S3. Compilation of SAR OSL measurements on multi grain quartz samples. The Central Age Model (Galbraith et al. 1999) was used for age calculation of the data set with overdispersion values < 25%. The three parametric Minimum Age Model (MAM3, Galbraith et al. 1999) was applied to samples with overdispersion values > 25%. Age results are given at 1 σ.

| Sample name | Lab.- Nr. | Age model | Grain size  (µm) | # discs (n) | OD (%) | De (Gy) | **Annual dose rate (Gy/ka)** | **Age (ka)** |
| --- | --- | --- | --- | --- | --- | --- | --- | --- |
| BDP2019-N5E1-U1-OSL1905 | LM19128-05 | MAM | 90-125 | 21 | 41.0 ± 1.4 | 2.6 ± 0.2 | **2.1 ± 0.1** | **1.2 ± 0.1** |
| BDP2019-N5E1-U2-OSL1907 | LM19128-07 | MAM | 90-125 | 23 | 33.4 ± 1.0 | 4.0 ± 0.2 | **1.9 ± 0.1** | **2.1 ± 0.2** |
| BDP2019-N5E1-U3-OSL1902 | LM19128-02 | MAM | 90-125 | 23 | 31.4 ± 1.0 | 9.8 ± 0.6 | **1.1 ± 0.1** | **8.9 ± 0.9** |
| BDP2019-N5E1-U5-OSL1906 | LM19128-06 | CAM | 90-125 | 19 | 18.0 ± 0.7 | 15.0 ± 0.6 | **2.3 ± 0.1** | **6.6 ± 0.4** |
| BDP2019-N5E1-U6-OSL1903 | LM19128-03 | CAM | 90-125 | 23 | 15.2 ± 0.5 | 16.7 ± 0.5 | **2.2 ± 0.1** | **7.6 ± 0.5** |
| BDP2019-N3E1-U7-OSL1904 | LM19128-04 | CAM | 90-125 | 21 | 19.6 ± 0.7 | 29.9 ± 1.3 | **2.1 ± 0.1** | **14.2 ± 1.1** |
| BDP2019-N4E1-U8-OSL1908 | LM19128-08 | CAM | 90-125 | 22 | 18.4 ± 0.6 | 42.6 ± 1.7 | **2.2 ± 0.1** | **19.4 ± 1.3** |
| BDP2019-N5E2-U9-OSL1901 | LM19128-01 | CAM | 90-125 | 15 | 24.7 ± 1.2 | 42.0 ± 1.4 | **2.4 ± 0.1** | **17.5 ± 1.0** |
| BDP2019-N4E1-U10-OSL1909 | LM19128-09 | MAM | 90-125 | 22 | 39.2 ± 1.3 | 48.3 ± 3.9 | **2.0 ± 0.1** | **24.8 ± 2.4** |

Table S4. Measurement protocol used for multi grain measurements on quartz. Multi grain measurements were made on small (2 mm) aliquots for De determination and dose recovery tests.

| Step | Treatment | Observed |
| --- | --- | --- |
| 1 | Natural/Regenerative dose |  |
| 2 | Preheat at 220-260°C for 10 s |  |
| 3 | Optically stimulate at 125 °C for 40 s | L_x_ |
| 4 | Give Test dose |  |
| 5 | Cut-Heat at 200-240 °C for 10 s |  |
| 6 | Optically stimulate at 125 °C for 40 s | T_x_ |
| 7 | Signal depletion: Optically stimulate at 250 °C for 40 s |  |
| 8 | Return to step 1 |  |

Table S5. Measurement protocol used in the portable reader (POSL) measurements.

| Step | Stimulation | Time (s) |
| --- | --- | --- |
| 1 | Dark | 15 |
| 2 | IR | 30 |
| 3 | IR | 30 |
| 4 | Dark | 15 |
| 5 | Blue OSL | 30 |
| 6 | Blue OSL | 30 |
| 7 | Dark | 15 |


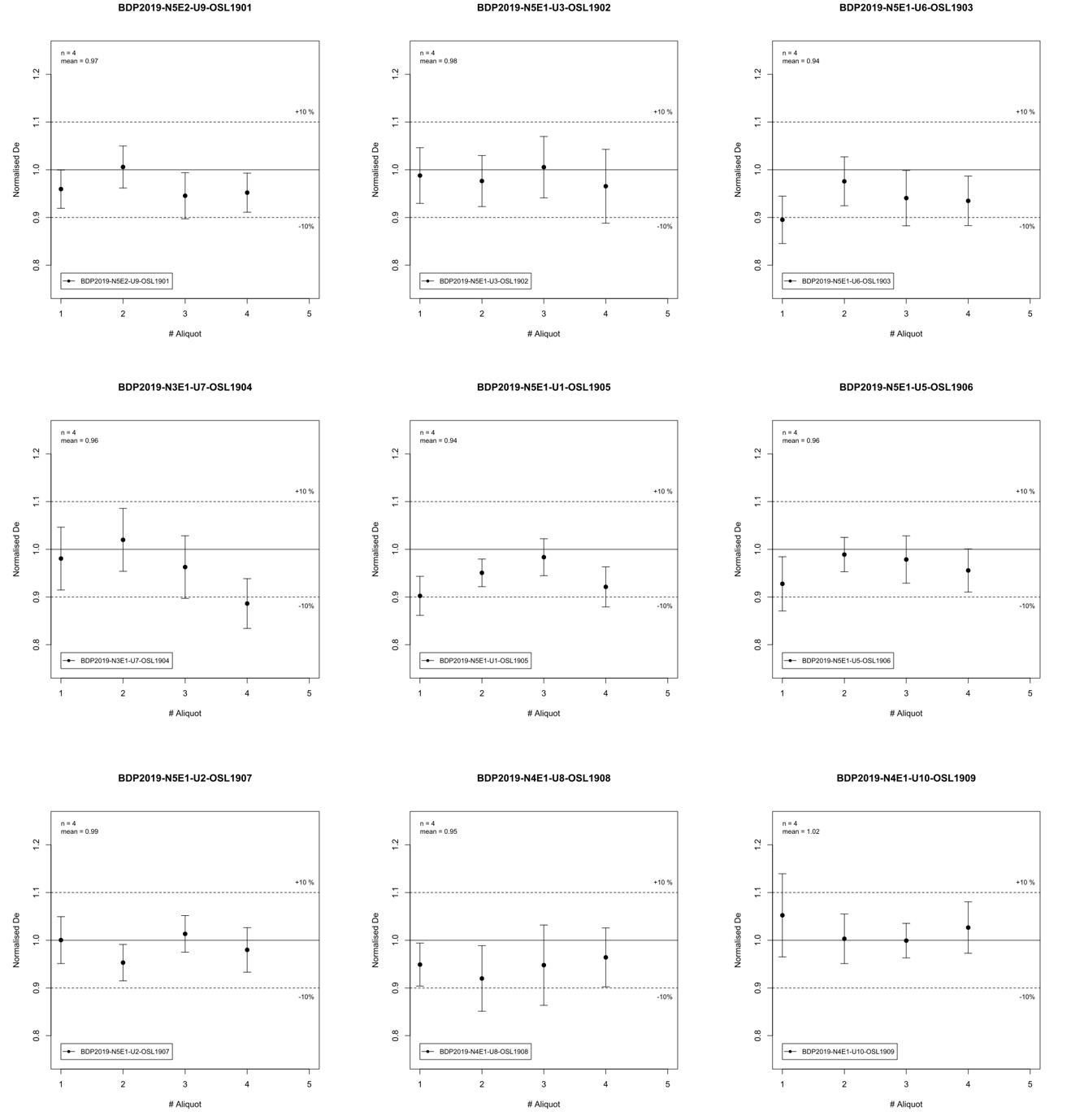


Fig. S2 Dose recovery test results.


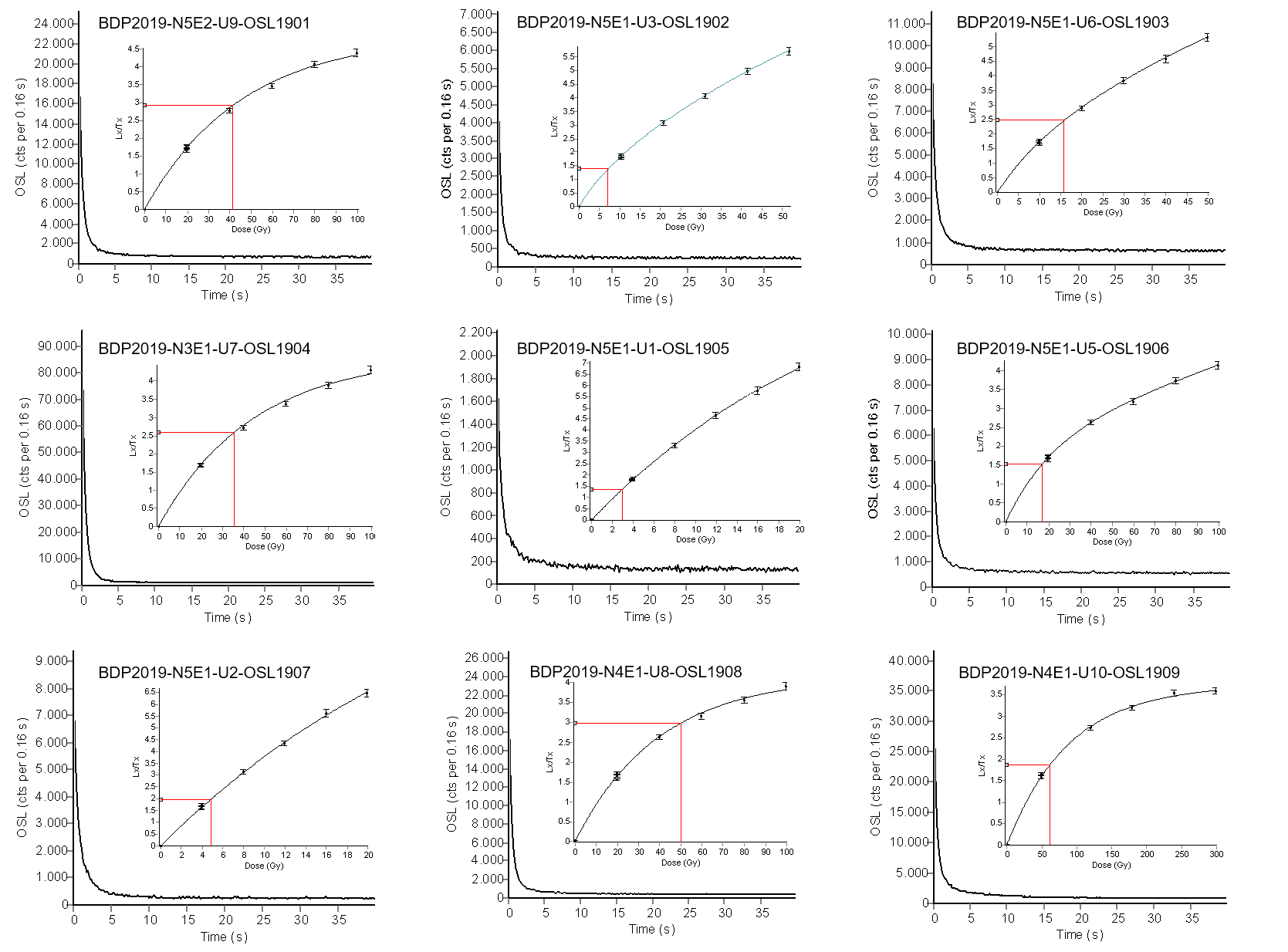


Fig. S3 Shine down curves and dose response curves (inset). Dose response curves have been fitted by a sum of two exponentials function. Open circle shows the sensitivity-corrected natural signal (L_n_/T_n_), while filled squares are the sensitivity-corrected regenerated points.


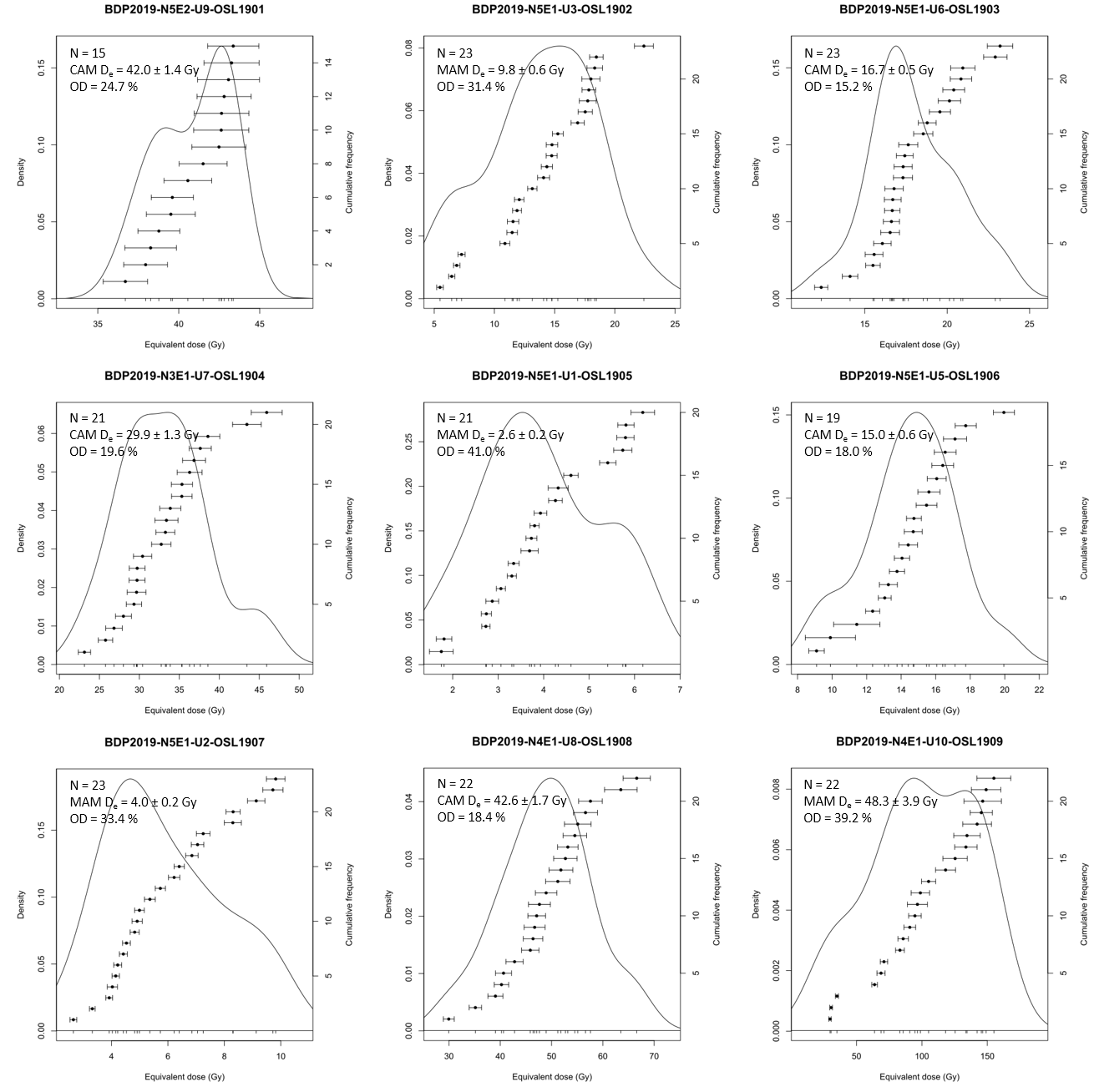


Fig. S4 Luminescence dating results. Multiple-grain equivalent dose (D_e_) distributions displayed as Kernell Density Estimate (KDE) plots. N= number of aliquots passing rejection criteria. OD = Overdispersion. MAM = Minimum Age Model. CAM = Central Age Model.

***Lithic Analyses***

Fig. S5 Raw material proportions for the total Pathirajawela excavation.

Fig. S6 Raw material proportions by unit for Pathirajawela

Fig. S7 Proportion of different artefact types in the Pathirajawela excavated assemblage.

Fig. S8 Average weight of artefacts in each unit.


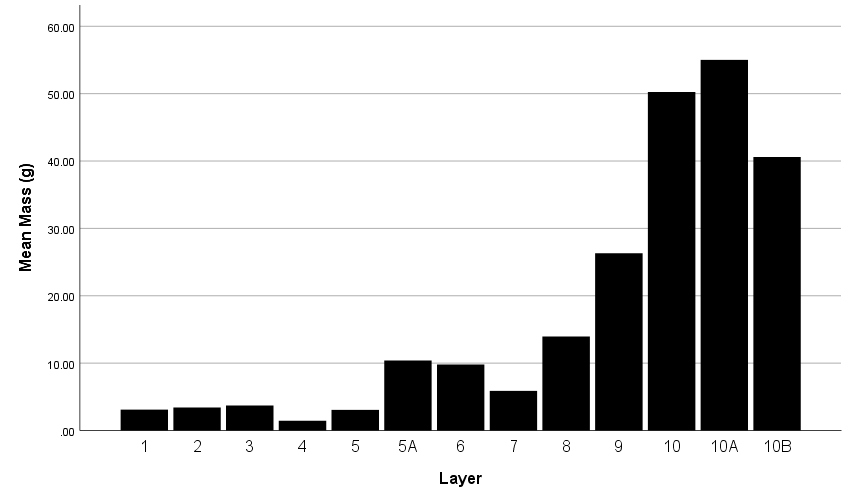


Fig. S9 Changes in mean mass for complete flakes by unit.


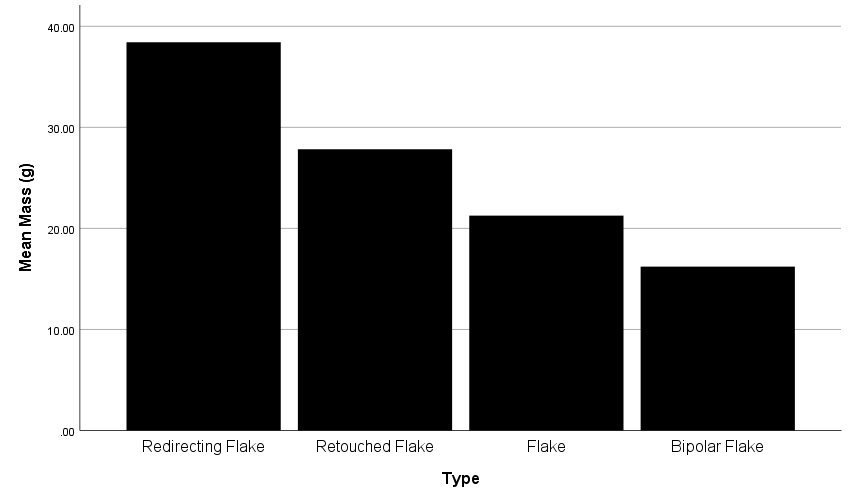


Fig. S10 Difference in the mass of flake types at Pathirajawela.

Fig. S11 Differences in the proportion of each core type in the Pathirajawela assemblage.


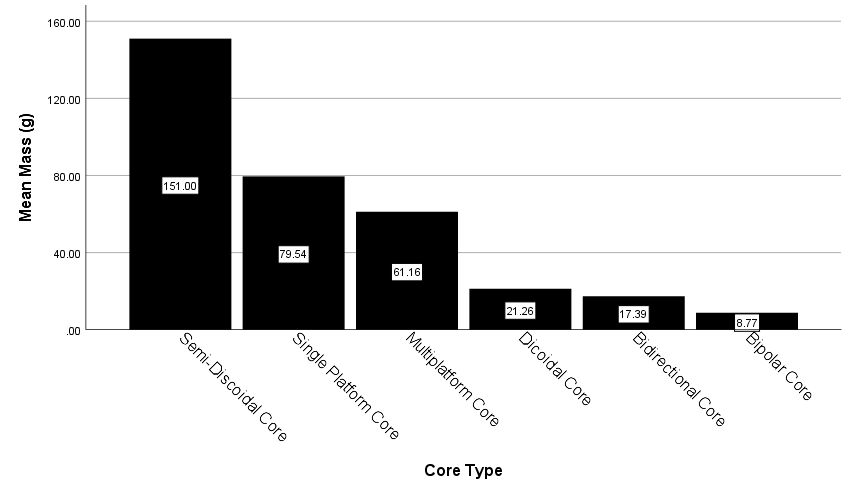


Fig. S12 Differences in mean mass of each core type in the Pathirajawela assemblage.


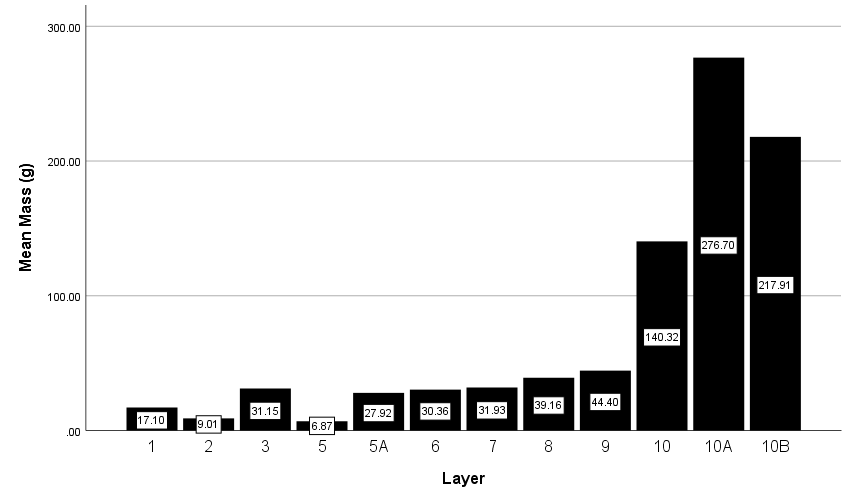


Fig. S13 Changing average core mass through time at Pathirajawela

Fig. S14 Changing proportions of core types by unit at Pathirajawela

Fig. S15 Changes in retouched flake types as a proportion of the retouched assemblage by unit at Pathirajawela

Table S6. Total numbers and weight of raw materials by context for Pathirajawela

| Context | Clear Quartz | g | Milky Quartz | g | Veiny Quartz | g | Chert | g | Quartzite | g | Pinky Quartz | g | Rose Quartz | g | Sandstone | g |
| --- | --- | --- | --- | --- | --- | --- | --- | --- | --- | --- | --- | --- | --- | --- | --- | --- |
| 1 | 288 | 85.9 | 238 | 402.31 | 15 | 72.39 | 0 | 0 | 0 | 0 | 3 | 7.98 | 0 | 0 | 0 | 0 |
| 2 | 434 | 248.09 | 165 | 278.93 | 8 | 7.72 | 0 | 0 | 1 | 42.47 | 2 | 0.28 | 0 | 0 | 0 | 0 |
| 3 | 387 | 506.603 | 175 | 765.06 | 15 | 37.9 | 1 | 0.32 | 0 | 0 | 0 | 0 | 10 | 4.03 | 4 | 72.03 |
| 4 | 27 | 314.6 | 6 | 12.44 | 1 | 7.44 | 0 | 0 | 0 | 0 | 0 | 0 | 0 | 0 | 0 | 0 |
| 5 | 12 | 26.11 | 8 | 55.64 | 0 | 0 | 0 | 0 | 0 | 0 | 0 | 0 | 0 | 0 | 0 | 0 |
| 5A | 95 | 836.4 | 54 | 629.99 | 6 | 47.29 | 0 | 0 | 0 | 0 | 0 | 0 | 0 | 0 | 0 | 0 |
| 6 | 109 | 1185.17 | 123 | 1580.61 | 7 | 321.25 | 0 | 0 | 0 | 0 | 0 | 0 | 2 | 4.21 | 0 | 0 |
| 7 | 1206 | 1105.42 | 360 | 2318.28 | 12 | 29.26 | 1 | 2.71 | 1 | 21.8 | 0 | 0 | 0 | 0 | 0 | 0 |
| 8 | 8 | 84.53 | 43 | 1117.03 | 2 | 23.58 | 0 | 0 | 0 | 0 | 0 | 0 | 0 | 0 | 0 | 0 |
| 9 | 14 | 335.47 | 87 | 2824.08 | 1 | 123.95 | 1 | 13.18 | 0 | 0 | 0 | 0 | 0 | 0 | 0 | 0 |
| 10 | 30 | 240.65 | 289 | 11024.84 | 104 | 5457.621 | 4 | 754.89 | 1 | 352.17 | 0 | 0 | 0 | 0 | 0 | 199.1 |
| 10a | 29 | 181.11 | 216 | 7388.03 | 90 | 5200.64 | 10 | 105.8 | 0 | 0 | 0 | 0 | 0 | 0 | 0 | 0 |
| 10b | 51 | 346.03 | 63 | 2725.13 | 20 | 2498.8 | 0 | 0 | 1 | 175 | 0 | 0 | 0 | 0 | 0 | 0 |
| Total | 2690 | 5496.083 | 1827 | 31122.37 | 281 | 13827.841 | 17 | 876.9 | 4 | 591.44 | 5 | 8.26 | 12 | 8.24 | 4 | 271.13 |

Table S7. Total count and proportions of artefact types in the Pathirajawela excavated assemblage.

| **Context** | **Complete Flake** | **%** | **Broken Flake** | **%** | **Flaked Piece** | **%** | **Redirecting Flake** | **%** | **Bipolar** | **%** | **Cores** | **%** | **Hammerstones** | **%** | **Retouched** | **%** | **Total** | **Density m3** |
| --- | --- | --- | --- | --- | --- | --- | --- | --- | --- | --- | --- | --- | --- | --- | --- | --- | --- | --- |
| 1 | 59 | 10.8 | 127 | 23.3 | 352 | 64.6 | 1 | 0.2 | 0 | 0.0 | 5 | 0.9 | 0 | 0.0 | 1 | 0.2 | 545 | 77.3 |
| 2 | 86 | 14.1 | 217 | 35.6 | 287 | 47.0 | 2 | 0.3 | 1 | 0.2 | 11 | 1.8 | 1 | 0.2 | 5 | 0.8 | 610 | 172 |
| 3 | 78 | 12.9 | 206 | 34.2 | 280 | 46.4 | 0 | 0.0 | 8 | 1.3 | 18 | 3.0 | 0 | 0.0 | 13 | 2.2 | 603 | 412 |
| 4 | 7 | 20.6 | 15 | 44.1 | 9 | 26.5 | 0 | 0.0 | 0 | 0.0 | 3 | 8.8 | 0 | 0.0 | 0 | 0.0 | 34 | 306 |
| 5 | 2 | 9.5 | 4 | 19.0 | 10 | 47.6 | 1 | 4.8 | 0 | 0.0 | 3 | 14.3 | 0 | 0.0 | 1 | 4.8 | 21 | 304 |
| 5a | 41 | 26.1 | 48 | 30.6 | 36 | 22.9 | 2 | 1.3 | 2 | 1.3 | 18 | 11.5 | 0 | 0.0 | 10 | 6.4 | 157 | 32.1 |
| 6 | 44 | 18.1 | 85 | 35.0 | 70 | 28.8 | 0 | 0.0 | 3 | 1.2 | 36 | 14.8 | 0 | 0.0 | 5 | 2.1 | 243 | 38.1 |
| 7 | 123 | 7.8 | 345 | 21.8 | 1043 | 65.8 | 2 | 0.1 | 7 | 0.4 | 34 | 2.1 | 3 | 0.2 | 28 | 1.8 | 1585 | 354 |
| 8 | 5 | 9.4 | 10 | 18.9 | 3 | 5.7 | 1 | 1.9 | 0 | 0.0 | 12 | 22.6 | 0 | 0.0 | 22 | 41.5 | 53 | 797 |
| 9 | 15 | 14.4 | 23 | 22.1 | 13 | 12.5 | 4 | 3.8 | 2 | 1.9 | 16 | 15.4 | 0 | 0.0 | 31 | 29.8 | 104 | 157 |
| 10 | 78 | 18.1 | 140 | 32.5 | 148 | 34.3 | 2 | 0.5 | 1 | 0.2 | 47 | 10.9 | 4 | 0.9 | 11 | 2.6 | 431 | 77.3 |
| 10a | 80 | 23.2 | 134 | 38.8 | 84 | 24.3 | 4 | 1.2 | 1 | 0.3 | 30 | 8.7 | 0 | 0.0 | 12 | 3.5 | 345 | 172 |
| 10b | 40 | 29.4 | 34 | 25.0 | 42 | 30.9 | 0 | 0.0 | 0 | 0.0 | 15 | 11.0 | 4 | 2.9 | 1 | 0.7 | 136 | 412 |
| Total | 658 | 13.6 | 1395 | 28.9 | 2337 | 48.3 | 19 | 0.4 | 25 | 0.5 | 248 | 5.1 | 12 | 0.2 | 140 | 2.9 | 4834 | mean=292 |

Table S8. Summary statistics for complete fakes by unit.

| Unit | | Mass | Length | Proximal Width | Width | Distal Width | Thickness | Number Arises | Platform Width | Platform Thickness | Platform Angle | % Cortex | L:T | W:T | Marginal Angle | Widest Point:Length | Number of Dorsal Scars |
| --- | --- | --- | --- | --- | --- | --- | --- | --- | --- | --- | --- | --- | --- | --- | --- | --- | --- |
| 1 | Mean | 3.1 | 20.5 | 12.2 | 18.8 | 13.6 | 6.6 | 1.3 | 10.5 | 4.7 | 66.1 | 22.1 | 1.2 | 3.1 | -4.1 | 0.7 | 3.9 |
|  | N | 14 | 14 | 14 | 14 | 14 | 14 | 3 | 12 | 12 | 12 | 14 | 14 | 14 | 14 | 14 | 14 |
|  | SD | 2.1 | 4.5 | 4.0 | 5.3 | 7.5 | 2.3 | 0.6 | 4.5 | 2.4 | 9.3 | 34.5 | 0.5 | 1.0 | 22.3 | 0.2 | 2.8 |
| 2 | Mean | 3.4 | 20.3 | 12.9 | 16.7 | 13.6 | 6.8 | 1.5 | 12.0 | 12.1 | 66.7 | 15.3 | 1.3 | 2.6 | -3.4 | 0.5 | 4.2 |
|  | N | 15 | 15 | 15 | 15 | 15 | 15 | 2 | 13 | 13 | 12 | 15 | 15 | 15 | 15 | 15 | 15 |
|  | SD | 2.4 | 6.0 | 7.3 | 5.3 | 8.7 | 2.4 | 0.7 | 7.4 | 24.8 | 14.3 | 27.2 | 0.5 | 0.6 | 28.7 | 0.2 | 1.5 |
| 3 | Mean | 3.7 | 20.7 | 14.7 | 17.9 | 14.9 | 7.2 | 1.3 | 13.1 | 5.4 | 71.0 | 17.0 | 1.2 | 2.6 | -0.2 | 0.6 | 3.8 |
|  | N | 37 | 37 | 37 | 37 | 37 | 37 | 7 | 32 | 32 | 32 | 37 | 37 | 37 | 37 | 37 | 37 |
|  | SD | 2.8 | 6.8 | 6.0 | 4.2 | 7.7 | 2.5 | 0.5 | 6.7 | 2.9 | 11.5 | 27.7 | 0.5 | 0.6 | 23.4 | 0.3 | 1.7 |
| 4 | Mean | 1.5 | 20.2 | 11.4 | 13.6 | 4.1 | 6.1 |  | 10.9 | 4.0 | 62.0 | 60.0 | 1.5 | 2.2 | 20.6 | 0.5 | 1.0 |
|  | N | 1 | 1 | 1 | 1 | 1 | 1 |  | 1 | 1 | 1 | 1 | 1 | 1 | 1 | 1 | 1 |
| 5 | Mean | 3.1 | 20.5 | 11.0 | 13.8 | 13.4 | 8.1 |  | 12.7 | 6.4 | 86.0 | 15.0 | 1.5 | 1.7 | -10.0 | 0.6 | 3.0 |
|  | N | 2 | 2 | 2 | 2 | 2 | 2 |  | 1 | 1 | 1 | 2 | 2 | 2 | 2 | 2 | 2 |
|  | SD | 1.8 | 3.6 | 8.1 | 3.8 | 7.7 | 2.7 |  |  |  |  | 21.2 | 0.1 | 0.1 | 44.7 | 0.4 | 1.4 |
| 5a | Mean | 10.4 | 28.9 | 19.1 | 25.8 | 20.5 | 10.0 | 1.0 | 18.3 | 7.6 | 76.5 | 9.1 | 1.2 | 2.9 | -3.4 | 0.6 | 4.6 |
|  | N | 34 | 34 | 34 | 34 | 34 | 34 | 4 | 31 | 31 | 30 | 34 | 34 | 34 | 34 | 34 | 33 |
|  | SD | 7.4 | 7.2 | 7.1 | 6.5 | 10.1 | 4.4 | 0.0 | 7.5 | 4.1 | 8.8 | 23.9 | 0.4 | 0.9 | 22.2 | 0.2 | 2.0 |
| 6 | Mean | 9.8 | 28.5 | 18.9 | 25.0 | 19.2 | 9.4 | 1.3 | 18.0 | 7.4 | 74.1 | 27.4 | 1.2 | 2.9 | -1.0 | 0.6 | 3.7 |
|  | N | 43 | 43 | 43 | 43 | 43 | 43 | 6 | 39 | 39 | 39 | 43 | 43 | 43 | 43 | 43 | 42 |
|  | SD | 8.0 | 7.2 | 7.1 | 7.1 | 9.6 | 3.7 | 0.5 | 7.8 | 4.0 | 17.3 | 37.9 | 0.4 | 0.9 | 18.1 | 0.2 | 2.6 |
| 7 | Mean | 5.9 | 25.4 | 15.3 | 19.6 | 13.7 | 7.5 | 1.4 | 15.1 | 6.3 | 71.2 | 12.0 | 1.4 | 2.8 | 4.6 | 0.5 | 4.2 |
|  | N | 42 | 42 | 42 | 42 | 42 | 41 | 5 | 37 | 37 | 36 | 41 | 42 | 41 | 42 | 41 | 40 |
|  | SD | 5.8 | 6.7 | 6.3 | 7.4 | 7.9 | 3.4 | 0.5 | 6.4 | 3.2 | 10.7 | 27.2 | 0.5 | 0.8 | 21.0 | 0.2 | 1.9 |
| 8 | Mean | 14.0 | 32.7 | 19.4 | 27.5 | 22.4 | 12.1 | 1.0 | 16.5 | 6.9 | 69.8 | 18.3 | 1.3 | 2.4 | -3.7 | 0.6 | 5.0 |
|  | N | 6 | 6 | 6 | 6 | 6 | 6 | 1 | 6 | 6 | 6 | 6 | 6 | 6 | 6 | 6 | 5 |
|  | SD | 6.2 | 12.0 | 6.6 | 6.0 | 8.0 | 3.4 |  | 7.4 | 3.7 | 19.9 | 36.0 | 0.6 | 0.7 | 18.3 | 0.3 | 2.7 |
| 9 | Mean | 26.3 | 35.9 | 26.8 | 33.7 | 27.3 | 14.1 | 1.0 | 23.3 | 11.2 | 75.0 | 42.7 | 1.1 | 2.5 | 0.6 | 0.7 | 3.7 |
|  | N | 15 | 15 | 15 | 15 | 15 | 15 | 1 | 11 | 11 | 11 | 15 | 15 | 15 | 15 | 15 | 14 |
|  | SD | 19.0 | 10.4 | 8.6 | 9.1 | 14.5 | 5.1 |  | 9.7 | 5.6 | 12.4 | 40.1 | 0.3 | 0.7 | 21.2 | 0.2 | 1.9 |
| 10 | Mean | 50.2 | 38.7 | 30.4 | 38.2 | 29.8 | 15.3 | 1.0 | 29.7 | 11.6 | 76.5 | 20.3 | 1.1 | 2.7 | 1.0 | 0.6 | 3.9 |
|  | N | 36 | 36 | 36 | 36 | 36 | 36 | 3 | 29 | 29 | 28 | 35 | 36 | 36 | 36 | 35 | 35 |
|  | SD | 77.1 | 14.6 | 15.2 | 17.8 | 20.7 | 7.4 | 0.0 | 15.7 | 7.8 | 11.1 | 35.0 | 0.5 | 0.8 | 24.9 | 0.2 | 2.8 |
| 10a | Mean | 55.0 | 35.4 | 32.2 | 39.2 | 31.4 | 16.6 | 1.0 | 33.2 | 15.7 | 76.3 | 12.2 | 1.0 | 2.6 | 2.2 | 0.6 | 3.8 |
|  | N | 32 | 32 | 32 | 32 | 32 | 32 | 3 | 28 | 28 | 28 | 32 | 32 | 32 | 32 | 32 | 32 |
|  | SD | 96.4 | 14.0 | 17.8 | 22.4 | 23.3 | 10.6 | 0.0 | 17.2 | 10.1 | 11.8 | 25.0 | 0.4 | 1.1 | 28.3 | 0.3 | 2.2 |
| 10b | Mean | 40.6 | 36.5 | 21.1 | 31.3 | 24.4 | 11.4 | 1.3 | 17.2 | 8.4 | 77.2 | 10.7 | 1.3 | 3.1 | -2.7 | 0.5 | 4.3 |
|  | N | 30 | 30 | 30 | 30 | 30 | 30 | 3 | 27 | 27 | 25 | 29 | 30 | 30 | 30 | 29 | 29 |
|  | SD | 68.6 | 16.5 | 9.5 | 17.5 | 17.3 | 6.9 | 0.6 | 7.3 | 7.8 | 9.1 | 24.6 | 0.5 | 1.2 | 21.5 | 0.3 | 1.9 |
| Total | Mean | 21.3 | 29.5 | 20.6 | 26.8 | 20.9 | 10.6 | 1.2 | 19.3 | 8.7 | 73.6 | 17.8 | 1.2 | 2.8 | -0.2 | 0.6 | 4.0 |
|  | N | 307 | 307 | 307 | 307 | 307 | 306 | 38 | 267 | 267 | 261 | 304 | 307 | 306 | 307 | 304 | 299 |
|  | SD | 50.0 | 12.0 | 11.8 | 14.4 | 15.2 | 6.5 | 0.4 | 11.9 | 8.3 | 12.5 | 31.0 | 0.5 | 0.9 | 22.7 | 0.3 | 2.2 |

Table S9. Comparison of means between upper (Units 1-7) and lower (Units 8-10B) Units at Pathirajawela. Non-significant results shown in bold.

| Attribute | | N | Mean | Std. Deviation | Std. Error Mean | t | df | Sig. (2-tailed) |
| --- | --- | --- | --- | --- | --- | --- | --- | --- |
| Mass | Upper | 188 | 6.71 | 6.52 | 0.48 | -5.49 | 119.15 | <0.0005 |
|  | Lower | 119 | 44.25 | 74.42 | 6.82 |  |  |  |
| Length | Upper | 188 | 24.97 | 7.52 | 0.55 | -8.19 | 160.05 | <0.0005 |
|  | Lower | 119 | 36.58 | 14.26 | 1.31 |  |  |  |
| Proximal Width | Upper | 188 | 16.23 | 6.90 | 0.50 | -8.02 | 152.80 | <0.0005 |
|  | Lower | 119 | 27.54 | 14.38 | 1.32 |  |  |  |
| Width | Upper | 188 | 21.25 | 7.14 | 0.52 | -8.27 | 141.59 | <0.0005 |
|  | Lower | 119 | 35.62 | 18.08 | 1.66 |  |  |  |
| Distal Width | Upper | 188 | 16.36 | 9.08 | 0.66 | -6.20 | 150.78 | <0.0005 |
|  | Lower | 119 | 28.17 | 19.51 | 1.79 |  |  |  |
| Thickness | Upper | 187 | 8.18 | 3.56 | 0.26 | -7.86 | 147.40 | <0.0005 |
|  | Lower | 119 | 14.36 | 8.10 | 0.74 |  |  |  |
| Number Arises | Upper | 27 | 1.30 | 0.47 | 0.09 | 1.61 | 28.50 | **0.119** |
|  | Lower | 11 | 1.09 | 0.30 | 0.09 |  |  |  |
| Platform Width | Upper | 166 | 15.38 | 7.33 | 0.57 | -6.60 | 130.13 | <0.0005 |
|  | Lower | 101 | 25.83 | 14.85 | 1.48 |  |  |  |
| Platform Thickness | Upper | 166 | 6.94 | 7.67 | 0.60 | -4.43 | 193.67 | <0.0005 |
|  | Lower | 101 | 11.54 | 8.55 | 0.85 |  |  |  |
| Platform Angle | Upper | 163 | 72.15 | 12.88 | 1.01 | -2.53 | 222.28 | 0.012 |
|  | Lower | 98 | 76.05 | 11.52 | 1.16 |  |  |  |
| % Cortex | Upper | 187 | 17.33 | 30.47 | 2.23 | -0.31 | 237.19 | **0.760** |
|  | Lower | 117 | 18.46 | 32.02 | 2.96 |  |  |  |
| W:T | Upper | 187 | 2.79 | 0.82 | 0.06 | 0.66 | 219.08 | **0.509** |
|  | Lower | 119 | 2.72 | 0.98 | 0.09 |  |  |  |
| Widest:Length | Upper | 187 | 0.58 | 0.25 | 0.02 | 0.07 | 227.13 | **0.948** |
|  | Lower | 117 | 0.58 | 0.27 | 0.03 |  |  |  |
| L:W | Upper | 188 | 1.27 | 0.47 | 0.03 | 2.19 | 252.67 | 0.029 |
|  | Lower | 119 | 1.15 | 0.47 | 0.04 |  |  |  |
| Marginal Angle | Upper | 188 | -0.44 | 21.94 | 1.60 | -0.20 | 234.08 | **0.842** |
|  | Lower | 119 | 0.10 | 24.05 | 2.20 |  |  |  |
| Number Dorsal Scars | Upper | 184 | 4.02 | 2.11 | 0.16 | 0.03 | 227.29 | **0.977** |
|  | Lower | 115 | 4.01 | 2.29 | 0.21 |  |  |  |

Table S10. Counts and proportion of platform preparation types for complete flakes by unit.

| Unit | None | Both | Faceting | Overhang Removal | Total |
| --- | --- | --- | --- | --- | --- |
| 1 | 9 | 1 | 0 | 4 | 14 |
|  | 64.3% | 7.1% | 0.0% | 28.6% | 100.0% |
| 2 | 6 | 2 | 2 | 5 | 15 |
|  | 40.0% | 13.3% | 13.3% | 33.3% | 100.0% |
| 3 | 10 | 1 | 4 | 22 | 37 |
|  | 27.0% | 2.7% | 10.8% | 59.5% | 100.0% |
| 4 | 0 | 0 | 0 | 1 | 1 |
|  | 0.0% | 0.0% | 0.0% | 100.0% | 100.0% |
| 5 | 1 | 0 | 0 | 1 | 2 |
|  | 50.0% | 0.0% | 0.0% | 50.0% | 100.0% |
| 5a | 14 | 3 | 5 | 12 | 34 |
|  | 41.2% | 8.8% | 14.7% | 35.3% | 100.0% |
| 6 | 13 | 0 | 1 | 29 | 43 |
|  | 30.2% | 0.0% | 2.3% | 67.4% | 100.0% |
| 7 | 21 | 3 | 0 | 18 | 42 |
|  | 50.0% | 7.1% | 0.0% | 42.9% | 100.0% |
| 8 | 3 | 0 | 0 | 3 | 6 |
|  | 50.0% | 0.0% | 0.0% | 50.0% | 100.0% |
| 9 | 9 | 1 | 0 | 5 | 15 |
|  | 60.0% | 6.7% | 0.0% | 33.3% | 100.0% |
| 10 | 27 | 0 | 0 | 9 | 36 |
|  | 75.0% | 0.0% | 0.0% | 25.0% | 100.0% |
| 10a | 22 | 1 | 1 | 8 | 32 |
|  | 68.8% | 3.1% | 3.1% | 25.0% | 100.0% |
| 10b | 9 | 1 | 2 | 18 | 30 |
|  | 30.0% | 3.3% | 6.7% | 60.0% | 100.0% |
| Total | 144 | 13 | 15 | 135 | 307 |
|  | 46.9% | 4.2% | 4.9% | 44.0% | 100.0% |

Table S11. Count and proportions of platform types for complete flakes by unit.

| Layer | Cortical | Cortical and Multiple | Cortical and Single | Crushed | Crushed Point | Crushed Ridge | Dihedral | Faceted Dihedral | Focalised | Multiple Scars | Single Scar | Total |
| --- | --- | --- | --- | --- | --- | --- | --- | --- | --- | --- | --- | --- |
| 1 | 6 | 0 | 0 | 1 | 0 | 1 | 0 | 0 | 0 | 3 | 3 | 14 |
|  | 42.9% | 0.0% | 0.0% | 7.1% | 0.0% | 7.1% | 0.0% | 0.0% | 0.0% | 21.4% | 21.4% | 100.0% |
| 2 | 2 | 1 | 0 | 1 | 0 | 1 | 0 | 0 | 0 | 4 | 6 | 15 |
|  | 13.3% | 6.7% | 0.0% | 6.7% | 0.0% | 6.7% | 0.0% | 0.0% | 0.0% | 26.7% | 40.0% | 100.0% |
| 3 | 6 | 1 | 0 | 0 | 0 | 5 | 0 | 0 | 2 | 8 | 15 | 37 |
|  | 16.2% | 2.7% | 0.0% | 0.0% | 0.0% | 13.5% | 0.0% | 0.0% | 5.4% | 21.6% | 40.5% | 100.0% |
| 4 | 0 | 0 | 0 | 0 | 0 | 0 | 0 | 0 | 0 | 0 | 1 | 1 |
|  | 0.0% | 0.0% | 0.0% | 0.0% | 0.0% | 0.0% | 0.0% | 0.0% | 0.0% | 0.0% | 100.0% | 100.0% |
| 5 | 0 | 0 | 0 | 0 | 1 | 0 | 0 | 0 | 0 | 0 | 1 | 2 |
|  | 0.0% | 0.0% | 0.0% | 0.0% | 50.0% | 0.0% | 0.0% | 0.0% | 0.0% | 0.0% | 50.0% | 100.0% |
| 5a | 2 | 1 | 1 | 0 | 0 | 3 | 1 | 1 | 0 | 13 | 12 | 34 |
|  | 5.9% | 2.9% | 2.9% | 0.0% | 0.0% | 8.8% | 2.9% | 2.9% | 0.0% | 38.2% | 35.3% | 100.0% |
| 6 | 8 | 0 | 0 | 0 | 2 | 3 | 2 | 0 | 1 | 2 | 25 | 43 |
|  | 18.6% | 0.0% | 0.0% | 0.0% | 4.7% | 7.0% | 4.7% | 0.0% | 2.3% | 4.7% | 58.1% | 100.0% |
| 7 | 2 | 0 | 0 | 1 | 1 | 2 | 0 | 0 | 1 | 10 | 23 | 42 |
|  | 4.8% | 0.0% | 0.0% | 2.4% | 2.4% | 4.8% | 0.0% | 0.0% | 2.4% | 23.8% | 54.8% | 100.0% |
| 8 | 0 | 0 | 0 | 0 | 0 | 0 | 1 | 0 | 1 | 2 | 2 | 6 |
|  | 0.0% | 0.0% | 0.0% | 0.0% | 0.0% | 0.0% | 16.7% | 0.0% | 16.7% | 33.3% | 33.3% | 100.0% |
| 9 | 1 | 0 | 0 | 0 | 0 | 4 | 1 | 0 | 0 | 3 | 6 | 15 |
|  | 6.7% | 0.0% | 0.0% | 0.0% | 0.0% | 26.7% | 6.7% | 0.0% | 0.0% | 20.0% | 40.0% | 100.0% |
| 10 | 5 | 0 | 0 | 1 | 0 | 4 | 3 | 0 | 0 | 2 | 20 | 36 |
|  | 13.9% | 0.0% | 0.0% | 2.8% | 0.0% | 11.1% | 8.3% | 0.0% | 0.0% | 5.6% | 55.6% | 100.0% |
| 10a | 2 | 0 | 0 | 0 | 2 | 2 | 1 | 0 | 0 | 7 | 18 | 32 |
|  | 6.3% | 0.0% | 0.0% | 0.0% | 6.3% | 6.3% | 3.1% | 0.0% | 0.0% | 21.9% | 56.3% | 100.0% |
| 10b | 4 | 0 | 0 | 0 | 0 | 3 | 2 | 0 | 1 | 5 | 13 | 30 |
|  | 13.3% | 0.0% | 0.0% | 0.0% | 0.0% | 10.0% | 6.7% | 0.0% | 3.3% | 16.7% | 43.3% | 100.0% |
| Total | 38 | 3 | 1 | 4 | 6 | 28 | 11 | 1 | 6 | 59 | 145 | 307 |
|  | 12.4% | 1.0% | 0.3% | 1.3% | 2.0% | 9.1% | 3.6% | 0.3% | 2.0% | 19.2% | 47.2% | 100.0% |

Table S12. Counts and proportions of dorsal scar orientations for complete flakes by unit.

| Layer | Bidirectional | Cortical | Proximal | Non-Proximal | Weakly Centripetal | Total |
| --- | --- | --- | --- | --- | --- | --- |
| 1 | 2 | 1 | 10 | 1 | 0 | 14 |
|  | 14.3% | 7.1% | 71.4% | 7.1% | 0.0% | 100.0% |
| 2 | 0 | 0 | 9 | 1 | 0 | 15 |
|  | 0.0% | 0.0% | 60.0% | 6.7% | 0.0% | 100.0% |
| 3 | 4 | 2 | 29 | 1 | 0 | 37 |
|  | 10.8% | 5.4% | 78.4% | 2.7% | 0.0% | 100.0% |
| 4 | 0 | 0 | 1 | 0 | 0 | 1 |
|  | 0.0% | 0.0% | 100.0% | 0.0% | 0.0% | 100.0% |
| 5 | 0 | 0 | 2 | 0 | 0 | 2 |
|  | 0.0% | 0.0% | 100.0% | 0.0% | 0.0% | 100.0% |
| 5a | 5 | 2 | 21 | 1 | 4 | 34 |
|  | 14.7% | 5.9% | 61.8% | 2.9% | 11.8% | 100.0% |
| 6 | 3 | 8 | 28 | 1 | 2 | 43 |
|  | 7.0% | 18.4% | 65.1% | 2.3% | 4.6% | 100.0% |
| 7 | 5 | 2 | 30 | 3 | 1 | 42 |
|  | 11.9% | 4.8% | 71.4% | 7.1% | 2.4% | 100.0% |
| 8 | 3 | 0 | 3 | 0 | 0 | 6 |
|  | 50.0% | 0.0% | 50.0% | 0.0% | 0.0% | 100.0% |
| 9 | 2 | 1 | 9 | 1 | 2 | 15 |
|  | 13.3% | 6.7% | 60.0% | 6.7% | 13.3% | 100.0% |
| 10 | 4 | 3 | 20 | 1 | 2 | 36 |
|  | 11.1% | 8.3% | 55.6% | 2.8% | 5.6% | 100.0% |
| 10a | 5 | 1 | 18 | 1 | 2 | 32 |
|  | 15.6% | 3.1% | 56.3% | 3.1% | 6.2&% | 100.0% |
| 10b | 1 | 0 | 24 | 1 | 2 | 30 |
|  | 3.3% | 0.0% | 80.0% | 3.3% | 6.7% | 100.0% |
| Total | 34 | 19 | 204 | 12 | 12 | 307 |
|  | 11.1% | 6.2% | 66.4% | 3.9% | 3.9% | 100.0% |

Table S13. Summary statistics for cores by type.

| Type | | Mass | Length | Width | Thickness | % Cortex | Number Scars | Number Rotations | Longest Face Length | Platform Thickness | Base Thickness | Platform Width | Non-Feather Terminations | Number Parallel Scars | Number Platform Quadrants Used | Final Platform Angle |
| --- | --- | --- | --- | --- | --- | --- | --- | --- | --- | --- | --- | --- | --- | --- | --- | --- |
| Bidirectional Core | Mean | 17.4 | 30.0 | 27.8 | 17.6 | 30.0 | 7.0 | 1.7 | 28.4 | 14.7 | 14.9 | 27.0 | 1.0 | 0.0 | 2.0 | 82.7 |
|  | N | 3 | 3 | 3 | 3 | 3 | 3 | 3 | 3 | 3 | 2 | 3 | 3 | 2 | 3 | 3 |
|  | SD | 13.4 | 10.3 | 6.7 | 2.5 | 26.5 | 6.1 | 0.6 | 9.1 | 5.8 | 4.0 | 11.4 | 0.0 | 0.0 | 1.0 | 19.1 |
| Bipolar Core | Mean | 8.8 | 24.4 | 22.1 | 12.7 | 30.0 | 6.0 | 0.2 | 23.8 | 6.2 | 8.8 | 18.0 | 1.2 | 0.3 | 2.6 |  |
|  | N | 5 | 5 | 5 | 5 | 5 | 5 | 5 | 5 | 1 | 1 | 4 | 5 | 4 | 5 |  |
|  | SD | 5.4 | 5.2 | 5.1 | 5.6 | 30.0 | 4.6 | 0.4 | 4.7 |  |  | 1.4 | 0.8 | 0.5 | 1.3 |  |
| Discoidal Core | Mean | 21.3 | 31.7 | 30.4 | 18.7 | 0.0 | 15.3 | 1.0 | 30.3 | 19.8 | 8.8 | 24.9 | 3.1 | 0.2 | 4.0 | 76.9 |
|  | N | 8 | 8 | 8 | 8 | 8 | 8 | 8 | 8 | 1 | 1 | 1 | 7 | 6 | 8 | 8 |
|  | SD | 15.2 | 7.6 | 8.3 | 6.2 | 0.0 | 5.3 | 0.0 | 6.5 |  |  |  | 2.0 | 0.4 | 0.0 | 10.0 |
| Multiplatform Core | Mean | 61.2 | 31.3 | 34.5 | 25.3 | 24.1 | 8.2 | 2.4 | 29.7 | 24.1 | 16.7 | 32.5 | 2.0 | 0.2 | 2.0 | 83.5 |
|  | N | 87 | 87 | 87 | 87 | 87 | 87 | 87 | 87 | 84 | 84 | 84 | 84 | 80 | 86 | 86 |
|  | SD | 98.7 | 14.3 | 20.5 | 12.0 | 26.5 | 4.4 | 1.5 | 13.1 | 13.3 | 12.5 | 19.0 | 1.8 | 0.6 | 1.0 | 9.4 |
| Semi-Discoidal Core | Mean | 151.0 | 46.0 | 39.2 | 26.0 | 19.3 | 10.4 | 1.1 | 41.4 | 18.2 | 27.7 | 29.0 | 2.3 | 0.0 | 3.5 | 80.3 |
|  | N | 14 | 14 | 14 | 14 | 14 | 14 | 13 | 14 | 1 | 2 | 1 | 11 | 10 | 13 | 14 |
|  | SD | 318.0 | 15.9 | 13.4 | 11.0 | 14.9 | 2.6 | 0.6 | 13.6 |  | 33.1 |  | 1.9 | 0.0 | 0.7 | 10.9 |
| Single Platform Core | Mean | 79.5 | 31.1 | 38.4 | 31.3 | 51.7 | 4.8 | 0.1 | 28.7 | 31.1 | 16.1 | 41.7 | 0.7 | 1.5 | 2.3 | 81.2 |
|  | N | 19 | 19 | 19 | 19 | 18 | 19 | 19 | 19 | 19 | 19 | 19 | 19 | 19 | 19 | 19 |
|  | SD | 112.8 | 14.9 | 18.9 | 16.8 | 23.1 | 2.5 | 0.2 | 15.4 | 17.9 | 9.8 | 19.0 | 1.0 | 6.2 | 1.0 | 11.6 |
| Total | Mean | 67.7 | 32.5 | 34.7 | 25.2 | 26.2 | 8.3 | 1.8 | 30.6 | 24.8 | 16.7 | 33.3 | 1.9 | 0.4 | 2.3 | 82.4 |
|  | N | 136 | 136 | 136 | 136 | 135 | 136 | 135 | 136 | 109 | 109 | 112 | 129 | 121 | 134 | 130 |
|  | SD | 136.9 | 14.5 | 18.7 | 12.6 | 26.7 | 4.7 | 1.6 | 13.4 | 14.3 | 12.3 | 18.8 | 1.8 | 2.5 | 1.1 | 10.2 |

Table S14. Count and proportions of core types by unit.

| Unit | Core Fragment | % | Single Platform Core | % | Multiplatform Core | % | Semi-Discoidal Core | % | Discoidal Core | % | Discoidal Core Fragment | % | Bidirectional Core | % | Bipolar Core | % | **Total** |
| --- | --- | --- | --- | --- | --- | --- | --- | --- | --- | --- | --- | --- | --- | --- | --- | --- | --- |
| 1 | 1 | 20.0 | 1 | 20.0 | 2 | 40.0 | 0 | 0.0 | 0 | 0.0 | 0 | 0.0 | 1 | 20.0 | 0 | 0.0 | 5 |
| 2 | 2 | 18.2 | 1 | 9.1 | 7 | 63.6 | 0 | 0.0 | 1 | 9.1 | 0 | 0.0 | 0 | 0.0 | 0 | 0.0 | 11 |
| 3 | 3 | 15.0 | 4 | 20.0 | 10 | 50.0 | 0 | 0.0 | 1 | 5.0 | 0 | 0.0 | 0 | 0.0 | 2 | 10.0 | 20 |
| 4 | 1 | 33.3 | 0 | 0.0 | 2 | 66.7 | 0 | 0.0 | 0 | 0.0 | 0 | 0.0 | 0 | 0.0 | 0 | 0.0 | 3 |
| 5 | 0 | 0.0 | 0 | 0.0 | 2 | 66.7 | 0 | 0.0 | 1 | 33.3 | 0 | 0.0 | 0 | 0.0 | 0 | 0.0 | 3 |
| 5A | 8 | 42.1 | 2 | 10.5 | 8 | 42.1 | 0 | 0.0 | 0 | 0.0 | 0 | 0.0 | 0 | 0.0 | 1 | 5.3 | 19 |
| 6 | 7 | 19.4 | 4 | 11.1 | 23 | 63.9 | 0 | 0.0 | 2 | 5.6 | 0 | 0.0 | 0 | 0.0 | 0 | 0.0 | 36 |
| 7 | 10 | 27.8 | 4 | 11.1 | 17 | 47.2 | 0 | 0.0 | 2 | 5.6 | 0 | 0.0 | 1 | 2.8 | 1 | 2.8 | 36 |
| 8 | 1 | 8.3 | 0 | 0.0 | 4 | 33.3 | 5 | 41.7 | 2 | 16.7 | 0 | 0.0 | 0 | 0.0 | 0 | 0.0 | 12 |
| 9 | 6 | 35.3 | 1 | 5.9 | 4 | 23.5 | 3 | 17.6 | 2 | 11.8 | 0 | 0.0 | 0 | 0.0 | 1 | 5.9 | 17 |
| 10 | 11 | 23.4 | 10 | 21.3 | 22 | 46.8 | 3 | 6.4 | 0 | 0.0 | 1 | 2.1 | 0 | 0.0 | 0 | 0.0 | 47 |
| 10A | 9 | 30.0 | 9 | 30.0 | 7 | 23.3 | 3 | 10.0 | 0 | 0.0 | 2 | 6.7 | 0 | 0.0 | 0 | 0.0 | 30 |
| 10B | 0 | 0.0 | 5 | 33.3 | 8 | 53.3 | 2 | 13.3 | 0 | 0.0 | 0 | 0.0 | 0 | 0.0 | 0 | 0.0 | 15 |
| Total | 59 | 23.3 | 41 | 16.2 | 114 | 45.1 | 16 | 6.3 | 11 | 4.3 | 3 | 1.2 | 1 | 0.4 | 5 | 2.0 | 253 |

Table S15. Summary statistics for cores by unit.

| Unit | | Mass | Length | Width | Thickness | % Cortex | Number Scars | Number Rotations | Face Length | Platform Thickness | Base Thickness | Platform Width | Number Non-Feather Scars | Number Parallel Scars | Platform Quadrants | Last Platform Angle |
| --- | --- | --- | --- | --- | --- | --- | --- | --- | --- | --- | --- | --- | --- | --- | --- | --- |
| 1 | Mean | 17.1 | 27.1 | 25.1 | 20.7 | 35.0 | 3.5 | 2.3 | 23.5 | 15.9 | 9.3 | 25.2 | 1.0 | 0.7 | 2.0 | 90.3 |
|  | N | 4 | 4 | 4 | 4 | 4 | 4 | 4 | 4 | 4 | 4 | 4 | 4 | 3 | 4 | 4 |
|  | SD | 14.4 | 14.0 | 6.9 | 6.7 | 31.1 | 1.3 | 2.1 | 5.7 | 7.3 | 7.3 | 13.8 | 0.8 | 1.2 | 0.8 | 8.4 |
| 2 | Mean | 9.0 | 20.0 | 20.3 | 16.1 | 21.1 | 5.0 | 2.2 | 19.1 | 14.6 | 12.0 | 17.9 | 1.6 | 0.4 | 1.6 | 81.9 |
|  | N | 9 | 9 | 9 | 9 | 9 | 9 | 9 | 9 | 8 | 8 | 8 | 9 | 8 | 9 | 9 |
|  | SD | 7.6 | 3.1 | 5.5 | 5.0 | 26.7 | 4.1 | 1.8 | 4.0 | 6.0 | 5.5 | 8.0 | 1.5 | 0.5 | 1.1 | 5.9 |
| 3 | Mean | 31.1 | 23.2 | 24.9 | 19.0 | 29.5 | 7.2 | 1.4 | 22.0 | 18.5 | 11.3 | 25.0 | 1.5 | 0.1 | 2.4 | 83.5 |
|  | N | 22 | 22 | 22 | 22 | 21 | 22 | 22 | 22 | 19 | 19 | 20 | 22 | 19 | 22 | 20 |
|  | SD | 55.8 | 10.5 | 15.4 | 8.8 | 28.9 | 4.1 | 1.2 | 9.9 | 9.8 | 6.9 | 16.0 | 1.4 | 0.5 | 1.2 | 8.9 |
| 5 | Mean | 6.9 | 23.9 | 19.6 | 13.5 | 0.0 | 15.0 | 1.7 | 21.7 | 14.6 | 9.9 | 20.6 | 2.0 | 0.0 | 3.0 | 75.3 |
|  | N | 3 | 3 | 3 | 3 | 3 | 3 | 3 | 3 | 3 | 3 | 3 | 3 | 3 | 3 | 3 |
|  | SD | 1.4 | 1.5 | 1.8 | 1.4 | 0.0 | 9.5 | 1.2 | 3.7 | 8.3 | 2.1 | 4.4 | 3.5 | 0.0 | 1.0 | 6.5 |
| 5a | Mean | 27.9 | 26.8 | 27.9 | 16.0 | 28.3 | 6.3 | 1.8 | 24.7 | 17.4 | 20.6 | 27.0 | 1.3 | 0.1 | 2.1 | 83.2 |
|  | N | 12 | 12 | 12 | 12 | 12 | 12 | 12 | 12 | 10 | 9 | 11 | 10 | 9 | 11 | 10 |
|  | SD | 37.3 | 6.6 | 6.5 | 5.1 | 28.9 | 4.0 | 1.6 | 7.4 | 8.2 | 24.3 | 9.8 | 1.6 | 0.3 | 0.9 | 12.1 |
| 6 | Mean | 30.4 | 31.5 | 29.8 | 22.9 | 21.1 | 8.4 | 2.2 | 29.5 | 25.2 | 13.9 | 30.7 | 2.6 | 0.1 | 2.1 | 79.3 |
|  | N | 18 | 18 | 18 | 18 | 18 | 18 | 18 | 18 | 17 | 17 | 17 | 17 | 16 | 18 | 18 |
|  | SD | 30.8 | 10.8 | 6.8 | 6.1 | 24.2 | 2.5 | 1.7 | 8.5 | 10.2 | 8.8 | 7.1 | 2.1 | 0.3 | 1.1 | 11.1 |
| 7 | Mean | 31.9 | 28.2 | 31.3 | 23.0 | 22.4 | 9.4 | 2.4 | 28.7 | 18.7 | 12.5 | 28.6 | 2.1 | 0.3 | 2.1 | 80.8 |
|  | N | 17 | 17 | 17 | 17 | 17 | 17 | 17 | 17 | 15 | 15 | 15 | 17 | 17 | 17 | 16 |
|  | SD | 28.6 | 12.4 | 7.4 | 7.3 | 25.4 | 5.7 | 1.7 | 11.2 | 7.3 | 8.6 | 9.2 | 1.9 | 0.7 | 1.1 | 9.6 |
| 8 | Mean | 39.2 | 40.5 | 33.5 | 22.7 | 22.7 | 10.5 | 1.9 | 37.3 | 27.5 | 17.0 | 30.5 | 2.1 | 0.4 | 2.8 | 84.6 |
|  | N | 11 | 11 | 11 | 11 | 11 | 11 | 11 | 11 | 4 | 4 | 4 | 8 | 7 | 11 | 11 |
|  | SD | 19.9 | 8.6 | 6.1 | 5.5 | 23.7 | 3.4 | 1.2 | 8.0 | 6.2 | 6.9 | 5.5 | 1.7 | 1.1 | 1.4 | 9.3 |
| 9 | Mean | 44.4 | 34.9 | 37.8 | 26.6 | 20.0 | 10.7 | 1.2 | 30.5 | 29.2 | 20.6 | 37.6 | 2.3 | 0.0 | 3.0 | 77.1 |
|  | N | 10 | 10 | 10 | 10 | 10 | 10 | 10 | 10 | 5 | 5 | 6 | 10 | 10 | 10 | 9 |
|  | SD | 19.0 | 11.5 | 9.5 | 7.6 | 22.6 | 5.6 | 1.4 | 7.7 | 5.8 | 8.2 | 12.6 | 2.2 | 0.0 | 1.1 | 11.9 |
| 10 | Mean | 140.3 | 41.8 | 52.1 | 41.4 | 33.8 | 8.2 | 1.6 | 42.1 | 40.1 | 25.0 | 53.1 | 1.3 | 2.1 | 2.5 | 85.8 |
|  | N | 13 | 13 | 13 | 13 | 13 | 13 | 13 | 13 | 12 | 12 | 12 | 13 | 13 | 13 | 13 |
|  | SD | 120.7 | 13.5 | 19.6 | 13.9 | 32.5 | 4.4 | 1.8 | 13.6 | 12.1 | 10.1 | 17.8 | 1.5 | 7.5 | 1.1 | 11.9 |
| 10a | Mean | 276.7 | 62.1 | 73.4 | 47.8 | 41.1 | 7.4 | 1.4 | 55.7 | 48.4 | 32.8 | 70.6 | 1.8 | 0.0 | 2.0 | 84.6 |
|  | N | 9 | 9 | 9 | 9 | 9 | 9 | 8 | 9 | 6 | 7 | 6 | 8 | 8 | 8 | 9 |
|  | SD | 177.2 | 14.4 | 32.0 | 12.7 | 27.1 | 3.6 | 1.4 | 15.0 | 23.8 | 18.3 | 33.9 | 1.9 | 0.0 | 1.3 | 8.7 |
| 10b | Mean | 217.9 | 35.3 | 43.3 | 32.8 | 28.8 | 10.0 | 1.1 | 33.3 | 35.4 | 21.4 | 44.7 | 2.3 | 0.1 | 2.4 | 83.4 |
|  | N | 8 | 8 | 8 | 8 | 8 | 8 | 8 | 8 | 6 | 6 | 6 | 8 | 8 | 8 | 8 |
|  | SD | 410.7 | 9.8 | 14.4 | 14.2 | 27.0 | 5.3 | 1.6 | 12.1 | 18.8 | 3.4 | 16.4 | 1.5 | 0.4 | 0.9 | 12.4 |
| Total | Mean | 67.7 | 32.5 | 34.7 | 25.2 | 26.2 | 8.3 | 1.8 | 30.6 | 24.8 | 16.7 | 33.3 | 1.9 | 0.4 | 2.3 | 82.4 |
|  | N | 136 | 136 | 136 | 136 | 135 | 136 | 135 | 136 | 109 | 109 | 112 | 129 | 121 | 134 | 130 |
|  | SD | 136.9 | 14.5 | 18.7 | 12.6 | 26.7 | 4.7 | 1.6 | 13.4 | 14.3 | 12.3 | 18.8 | 1.8 | 2.5 | 1.1 | 10.2 |

Table S16. Comparison of mean differences in core attributes between the upper (Units 1-7) and lower (Units 8-10B) assemblages. Non-Significant differences are shown in bold.

| Attribute | | N | Mean | Std. Deviation | Std. Error Mean | t | df | Sig. (2-tailed) |
| --- | --- | --- | --- | --- | --- | --- | --- | --- |
| Mass | Upper | 85 | 26.8 | 37.3 | 4.0 | -3.82 | 52.1 | <0.0005 |
|  | Lower | 51 | 135.9 | 201.7 | 28.2 |  |  |  |
| Length | Upper | 85 | 26.3 | 10.4 | 1.1 | -6.95 | 79.6 | <0.0005 |
|  | Lower | 51 | 42.7 | 14.8 | 2.1 |  |  |  |
| Width | Upper | 85 | 26.9 | 10.2 | 1.1 | -6.23 | 62.6 | <0.0005 |
|  | Lower | 51 | 47.6 | 22.4 | 3.1 |  |  |  |
| Thickness | Upper | 85 | 19.8 | 7.3 | 0.8 | -6.72 | 66.0 | <0.0005 |
|  | Lower | 51 | 34.2 | 14.3 | 2.0 |  |  |  |
| % Cortex | Upper | 84 | 24.4 | 26.5 | 2.9 | -1.01 | 103.6 | **0.315** |
|  | Lower | 51 | 29.2 | 27.1 | 3.8 |  |  |  |
| Number Scars | Upper | 85 | 7.6 | 4.7 | 0.5 | -2.13 | 109.0 | 0.036 |
|  | Lower | 51 | 9.4 | 4.5 | 0.6 |  |  |  |
| Number Rotations | Upper | 85 | 2.0 | 1.6 | 0.2 | 1.80 | 108.5 | **0.075** |
|  | Lower | 50 | 1.5 | 1.5 | 0.2 |  |  |  |
| Longest Face Length | Upper | 85 | 25.1 | 9.3 | 1.0 | -6.67 | 76.8 | <0.0005 |
|  | Lower | 51 | 39.8 | 14.0 | 2.0 |  |  |  |
| Platform Thickness | Upper | 76 | 19.2 | 9.1 | 1.0 | -6.23 | 41.5 | <0.0005 |
|  | Lower | 33 | 37.6 | 15.8 | 2.8 |  |  |  |
| Base Thickness | Upper | 75 | 13.2 | 11.0 | 1.3 | -4.75 | 60.9 | <0.0005 |
|  | Lower | 34 | 24.4 | 11.6 | 2.0 |  |  |  |
| Platform Width | Upper | 78 | 26.3 | 11.4 | 1.3 | -5.64 | 40.6 | <0.0005 |
|  | Lower | 34 | 49.3 | 22.5 | 3.9 |  |  |  |
| Non-Feather | Upper | 82 | 1.8 | 1.8 | 0.2 | -0.16 | 98.4 | **0.872** |
|  | Lower | 47 | 1.9 | 1.7 | 0.3 |  |  |  |
| Number Parallel Scars | Upper | 75 | 0.2 | 0.5 | 0.1 | -0.82 | 45.9 | **0.415** |
|  | Lower | 46 | 0.7 | 4.0 | 0.6 |  |  |  |
| Platform Quadrants Used | Upper | 84 | 2.1 | 1.1 | 0.1 | -2.04 | 95.7 | 0.044 |
|  | Lower | 50 | 2.6 | 1.2 | 0.2 |  |  |  |
| Final Platform Angle | Upper | 80 | 81.8 | 9.7 | 1.1 | -0.81 | 95.3 | **0.419** |
|  | Lower | 50 | 83.4 | 10.9 | 1.5 |  |  |  |
| L:W | Upper | 85 | 1.0 | 0.3 | 0.0 | 0.51 | 91.8 | **0.608** |
|  | Lower | 51 | 1.0 | 0.3 | 0.0 |  |  |  |

Table S17. Retouched flake typology and counts by layer at Pathirajawela.

| Unit | 1 | 2 | 3 | 5 | 5a | 6 | 7 | 8 | 9 | 10 | 10a | 10b | Total |
| --- | --- | --- | --- | --- | --- | --- | --- | --- | --- | --- | --- | --- | --- |
| Asymmetric Backed |  |  | 1 |  |  |  |  |  |  |  |  |  | 1 |
| Bifacial Double Side and End Scraper |  |  |  |  |  |  | 1 |  |  |  |  |  | 1 |
| Denticulate |  |  |  |  | 1 | 1 |  |  | 1 |  |  |  | 3 |
| Double Side and Double End Scraper |  |  |  |  |  |  |  |  | 1 |  |  |  | 1 |
| Double Side and End Scraper |  | 1 | 1 |  |  | 1 | 1 |  | 2 | 1 |  |  | 7 |
| Double Side Scraper (Limace) |  |  |  |  |  |  | 1 |  |  |  |  |  | 1 |
| End Scraper |  | 1 | 3 | 1 | 1 |  | 2 | 4 | 1 |  | 1 |  | 14 |
| Nosed End Scraper |  |  |  |  |  | 1 | 1 |  |  |  |  |  | 2 |
| Nosed Scraper |  |  |  |  |  |  | 1 |  |  |  |  |  | 1 |
| Notch | 1 |  |  |  | 3 | 1 | 2 | 4 | 11 | 3 | 2 |  | 27 |
| Notched |  |  |  |  |  |  |  |  | 2 | 3 |  |  | 5 |
| Notched Double Side and End Scraper |  |  |  |  |  |  | 1 | 1 | 1 |  |  |  | 3 |
| Notched Double Side Scraper |  |  |  |  |  | 1 |  |  | 1 |  |  |  | 2 |
| Notched Double Side Scraper (Limace) |  |  |  |  |  |  |  | 1 | 1 |  |  |  | 2 |
| Notched End Scraper |  | 1 | 2 |  |  |  | 3 | 2 |  |  |  | 1 | 9 |
| Notched Side and End |  |  |  |  | 1 |  |  | 1 |  |  |  |  | 2 |
| Notched Side and End Scraper |  |  |  |  | 1 |  | 1 | 1 | 2 |  |  |  | 5 |
| Notched Side Scraper |  |  |  |  | 1 |  |  | 1 |  |  |  |  | 2 |
| Side and End Scraper |  | 1 |  |  |  |  |  | 1 |  |  |  |  | 2 |
| Side Scraper | 1 | 1 | 1 |  | 1 |  | 1 | 2 | 1 |  |  |  | 8 |
| Symmetric Backed - Lunate |  |  | 2 |  |  |  | 1 |  |  |  |  |  | 3 |
| Symmetric Backed - Triangle |  | 1 |  |  |  |  |  |  |  |  |  |  | 1 |
| Total | 2 | 6 | 10 | 1 | 10 | 5 | 17 | 19 | 24 | 7 | 4 | 1 | 106 |

Table S18. Summary statistics for retouched flakes by layer at Pathirajawela.

| Unit | | Mass | Length | Proximal Width | Width | Distal Width | Thickness | Number Arises | Platform Width | Platform Thickness | Platform Angle | % Cortex | Number Retouched Segments | Number Notches | Marginal Angle | Invasiveness Index | Retouch Perimeter | Retouched Edge Curvature | Kuhn Index | Retouch Edge Angle |
| --- | --- | --- | --- | --- | --- | --- | --- | --- | --- | --- | --- | --- | --- | --- | --- | --- | --- | --- | --- | --- |
| 1 | Mean | 3.9 | 20.6 | 12.6 | 19.2 | 13.8 | 7.9 | 1.3 | 11.4 | 5.7 | 68.9 | 18.2 | 3.0 | 1.0 | -3.3 | 0.1 | 0.2 | 0.1 | 0.1 | 9.6 |
|  | N | 17 | 17 | 17 | 17 | 17 | 17 | 3 | 15 | 15 | 15 | 17 | 2 | 1 | 17 | 2 | 2 | 2 | 17 | 17 |
|  | SD | 3.3 | 4.2 | 5.0 | 5.9 | 7.5 | 4.1 | 0.6 | 5.9 | 4.1 | 12.2 | 32.3 | 2.8 |  | 20.1 | 0.1 | 0.2 | 0.3 | 0.2 | 27.5 |
| 2 | Mean | 4.2 | 20.4 | 12.7 | 17.8 | 13.6 | 7.8 | 1.5 | 12.2 | 10.1 | 69.7 | 12.1 | 3.7 | 1.5 | -3.7 | 0.2 | 1.0 | 0.3 | 0.2 | 16.0 |
|  | N | 24 | 24 | 24 | 24 | 24 | 24 | 2 | 20 | 20 | 19 | 24 | 6 | 2 | 24 | 6 | 6 | 6 | 24 | 24 |
|  | SD | 2.9 | 6.4 | 6.9 | 5.7 | 8.1 | 3.3 | 0.7 | 6.6 | 19.9 | 13.4 | 23.2 | 2.7 | 0.7 | 25.3 | 0.2 | 1.3 | 0.3 | 0.4 | 32.0 |
| 3 | Mean | 4.8 | 20.5 | 14.8 | 18.1 | 15.1 | 7.8 | 1.4 | 13.9 | 5.8 | 70.9 | 14.6 | 3.3 | 1.5 | 1.2 | 0.1 | 0.5 | 0.3 | 0.2 | 16.7 |
|  | N | 50 | 50 | 49 | 49 | 49 | 50 | 8 | 38 | 37 | 37 | 50 | 10 | 2 | 50 | 10 | 9 | 9 | 50 | 50 |
|  | SD | 5.5 | 7.0 | 7.4 | 6.5 | 8.3 | 3.3 | 0.5 | 7.9 | 3.4 | 11.7 | 25.7 | 1.7 | 0.7 | 26.1 | 0.1 | 0.1 | 0.2 | 0.3 | 34.1 |
| 4 | Mean | 1.5 | 20.2 | 11.4 | 13.6 | 4.1 | 6.1 |  | 10.9 | 4.0 | 62.0 | 60.0 |  |  | 20.6 |  |  |  | 0.0 | 0.0 |
|  | N | 1 | 1 | 1 | 1 | 1 | 1 |  | 1 | 1 | 1 | 1 |  |  | 1 |  |  |  | 1 | 1 |
|  | SD |  |  |  |  |  |  |  |  |  |  |  |  |  |  |  |  |  |  |  |
| 5 | Mean | 2.9 | 19.2 | 10.9 | 15.1 | 11.2 | 7.5 |  | 11.8 | 6.2 | 86.0 | 10.0 | 1.0 |  | -2.0 | 0.0 | 0.3 | 0.1 | 0.3 | 27.8 |
|  | N | 3 | 3 | 3 | 3 | 3 | 3 |  | 2 | 2 | 2 | 3 | 1 |  | 3 | 1 | 1 | 1 | 3 | 3 |
|  | SD | 1.3 | 3.3 | 5.7 | 3.5 | 6.7 | 2.1 |  | 1.3 | 0.3 | 0.0 | 17.3 |  |  | 34.6 |  |  |  | 0.5 | 48.1 |
| 5a | Mean | 12.0 | 28.7 | 19.6 | 26.9 | 21.4 | 11.4 | 1.0 | 17.6 | 7.9 | 73.7 | 7.8 | 2.7 | 1.3 | -3.8 | 0.1 | -0.7 | 0.0 | 0.1 | 16.8 |
|  | N | 46 | 46 | 46 | 46 | 45 | 46 | 5 | 38 | 38 | 36 | 46 | 10 | 7 | 46 | 10 | 10 | 10 | 46 | 46 |
|  | SD | 8.2 | 7.5 | 8.0 | 7.1 | 9.8 | 5.5 | 0.0 | 7.4 | 4.4 | 15.6 | 21.8 | 2.3 | 0.8 | 24.3 | 0.1 | 3.0 | 0.2 | 0.3 | 32.6 |
| 6 | Mean | 13.7 | 29.3 | 19.8 | 25.5 | 19.1 | 10.1 | 1.4 | 19.1 | 7.7 | 74.5 | 25.5 | 4.4 | 1.8 | 0.3 | 0.2 | 0.4 | 0.4 | 0.1 | 7.9 |
|  | N | 49 | 49 | 49 | 49 | 49 | 49 | 7 | 42 | 42 | 42 | 49 | 5 | 4 | 49 | 4 | 5 | 5 | 49 | 49 |
|  | SD | 25.5 | 8.2 | 9.4 | 8.6 | 11.1 | 5.0 | 0.5 | 11.0 | 4.8 | 16.8 | 36.3 | 2.7 | 1.0 | 20.0 | 0.1 | 0.3 | 0.5 | 0.2 | 23.9 |
| 7 | Mean | 11.3 | 25.6 | 16.7 | 21.1 | 14.9 | 9.0 | 1.4 | 16.7 | 7.3 | 71.3 | 8.7 | 4.5 | 1.7 | 4.5 | 0.2 | 0.5 | 0.4 | 0.1 | 20.3 |
|  | N | 65 | 65 | 65 | 65 | 65 | 64 | 5 | 53 | 53 | 52 | 63 | 17 | 10 | 65 | 17 | 17 | 17 | 65 | 65 |
|  | SD | 32.2 | 6.7 | 7.4 | 7.5 | 8.7 | 4.4 | 0.5 | 7.6 | 3.7 | 11.1 | 23.2 | 2.9 | 0.9 | 19.6 | 0.2 | 0.3 | 0.5 | 0.3 | 34.6 |
| 8 | Mean | 16.9 | 33.4 | 23.7 | 29.2 | 18.9 | 12.3 | 1.0 | 20.9 | 9.1 | 67.7 | 28.0 | 3.3 | 3.1 | 7.2 | 0.1 | 0.3 | 0.1 | 0.3 | 54.6 |
|  | N | 26 | 26 | 26 | 26 | 26 | 26 | 1 | 25 | 25 | 25 | 25 | 19 | 12 | 26 | 19 | 19 | 18 | 26 | 26 |
|  | SD | 8.3 | 8.1 | 8.5 | 7.2 | 11.8 | 2.7 |  | 10.1 | 3.8 | 15.0 | 38.5 | 2.0 | 6.5 | 23.6 | 0.1 | 0.1 | 0.2 | 0.3 | 37.6 |
| 9 | Mean | 34.7 | 39.4 | 27.9 | 35.4 | 27.7 | 16.3 | 1.0 | 24.6 | 12.7 | 71.1 | 24.0 | 4.0 | 1.3 | 0.6 | 0.1 | 0.3 | 0.4 | 0.3 | 43.1 |
|  | N | 42 | 42 | 42 | 42 | 42 | 42 | 5 | 32 | 32 | 32 | 42 | 24 | 20 | 42 | 24 | 24 | 24 | 42 | 42 |
|  | SD | 24.7 | 10.3 | 10.5 | 9.8 | 13.1 | 5.9 | 0.0 | 10.6 | 6.4 | 21.0 | 34.7 | 3.5 | 0.6 | 22.1 | 0.1 | 0.2 | 1.1 | 0.3 | 40.9 |
| 10 | Mean | 54.4 | 41.3 | 31.3 | 38.6 | 29.7 | 16.7 | 1.0 | 29.9 | 12.5 | 78.4 | 20.0 | 2.3 | 1.5 | 3.0 | 0.1 | 0.3 | 0.0 | 0.1 | 12.0 |
|  | N | 45 | 45 | 45 | 45 | 45 | 45 | 4 | 37 | 37 | 37 | 44 | 7 | 6 | 45 | 7 | 7 | 7 | 45 | 45 |
|  | SD | 72.4 | 17.3 | 14.2 | 16.9 | 20.1 | 8.1 | 0.0 | 14.4 | 7.5 | 12.3 | 35.2 | 1.3 | 0.5 | 24.8 | 0.1 | 0.2 | 0.3 | 0.2 | 28.6 |
| 10a | Mean | 56.2 | 37.1 | 33.1 | 40.0 | 30.4 | 17.6 | 1.0 | 33.4 | 15.2 | 76.7 | 13.4 | 1.3 | 1.0 | 4.2 | 0.0 | 0.1 | 0.2 | 0.0 | 6.9 |
|  | N | 41 | 41 | 41 | 41 | 41 | 41 | 3 | 36 | 36 | 36 | 41 | 4 | 3 | 41 | 4 | 4 | 4 | 41 | 41 |
|  | SD | 87.4 | 14.6 | 17.3 | 20.9 | 21.5 | 10.3 | 0.0 | 15.8 | 9.2 | 14.4 | 24.1 | 0.5 | 0.0 | 27.4 | 0.0 | 0.0 | 0.7 | 0.1 | 21.4 |
| 10b | Mean | 39.5 | 37.2 | 21.8 | 30.3 | 23.7 | 11.5 | 1.4 | 17.5 | 8.6 | 76.5 | 9.7 | 3.0 | 1.0 | -1.0 | 0.1 | 0.3 | 0.3 | 0.0 | 1.9 |
|  | N | 33 | 33 | 33 | 33 | 33 | 32 | 5 | 28 | 28 | 26 | 32 | 1 | 1 | 33 | 1 | 1 | 1 | 33 | 33 |
|  | SD | 65.6 | 15.9 | 9.9 | 17.1 | 16.6 | 6.8 | 0.5 | 7.4 | 7.7 | 9.7 | 23.6 |  |  | 21.4 |  |  |  | 0.0 | 11.0 |
| Total | Mean | 23.4 | 30.5 | 21.6 | 27.6 | 21.0 | 11.8 | 1.3 | 20.3 | 9.3 | 73.1 | 16.1 | 3.5 | 1.7 | 1.2 | 0.1 | 0.3 | 0.2 | 0.1 | 18.2 |
|  | N | 442 | 442 | 441 | 441 | 440 | 440 | 48 | 367 | 366 | 360 | 437 | 106 | 68 | 442 | 105 | 105 | 104 | 442 | 442 |
|  | SD | 46.6 | 12.7 | 12.1 | 13.8 | 14.5 | 6.8 | 0.4 | 12.0 | 7.8 | 14.4 | 29.6 | 2.6 | 2.8 | 23.2 | 0.1 | 1.0 | 0.6 | 0.3 | 33.5 |

Table S19. Comparison of means for retouched flakes between the upper (Units 1-7) and lower (Units 8-10B) assemblages. Non-significant results are in bold.

| Attribute | | N | Mean | Std. Deviation | Std. Error Mean | t | df | Sig. (2-tailed) |
| --- | --- | --- | --- | --- | --- | --- | --- | --- |
| Mass | Upper | 255 | 9.3 | 20.5 | 1.3 | -6.96 | 215.1 | <0.0005 |
|  | Lower | 187 | 42.5 | 62.8 | 4.6 |  |  |  |
| Length | Upper | 255 | 25.0 | 7.9 | 0.5 | -11.54 | 271.0 | <0.0005 |
|  | Lower | 187 | 38.1 | 14.1 | 1.0 |  |  |  |
| Proximal Width | Upper | 254 | 16.7 | 8.1 | 0.5 | -10.39 | 283.3 | <0.0005 |
|  | Lower | 187 | 28.2 | 13.4 | 1.0 |  |  |  |
| Width | Upper | 254 | 21.9 | 8.0 | 0.5 | -10.60 | 253.5 | <0.0005 |
|  | Lower | 187 | 35.4 | 16.0 | 1.2 |  |  |  |
| Distal Width | Upper | 253 | 16.6 | 9.6 | 0.6 | -7.15 | 265.3 | <0.0005 |
|  | Lower | 187 | 26.8 | 17.7 | 1.3 |  |  |  |
| Thickness | Upper | 254 | 9.2 | 4.5 | 0.3 | -9.62 | 276.6 | <0.0005 |
|  | Lower | 186 | 15.3 | 7.8 | 0.6 |  |  |  |
| Number Arises | Upper | 30 | 1.3 | 0.5 | 0.1 | 1.91 | 45.3 | 0.062 |
|  | Lower | 18 | 1.1 | 0.3 | 0.1 |  |  |  |
| Platform Width | Upper | 209 | 16.0 | 8.5 | 0.6 | -8.18 | 247.1 | <0.0005 |
|  | Lower | 158 | 26.0 | 13.6 | 1.1 |  |  |  |
| Platform Thickness | Upper | 208 | 7.4 | 7.3 | 0.5 | -5.79 | 328.8 | <0.0005 |
|  | Lower | 158 | 11.9 | 7.6 | 0.6 |  |  |  |
| Platform Angle | Upper | 204 | 72.1 | 13.6 | 1.0 | -1.54 | 312.1 | **0.125** |
|  | Lower | 156 | 74.5 | 15.3 | 1.2 |  |  |  |
| % Cortex | Upper | 253 | 14.2 | 27.6 | 1.7 | -1.57 | 359.6 | **0.117** |
|  | Lower | 184 | 18.8 | 31.9 | 2.4 |  |  |  |
| Number Retouched Segments | Upper | 51 | 3.7 | 2.5 | 0.4 | 0.67 | 104.0 | **0.507** |
|  | Lower | 55 | 3.3 | 2.7 | 0.4 |  |  |  |
| Number Notches | Upper | 26 | 1.5 | 0.8 | 0.2 | -0.47 | 47.8 | **0.640** |
|  | Lower | 42 | 1.8 | 3.5 | 0.5 |  |  |  |
| Marginal Angle | Upper | 255 | 0.3 | 22.6 | 1.4 | -1.03 | 387.3 | **0.305** |
|  | Lower | 187 | 2.6 | 24.0 | 1.8 |  |  |  |
| Invasiveness Index | Upper | 50 | 0.2 | 0.1 | 0.0 | 1.45 | 92.4 | **0.152** |
|  | Lower | 55 | 0.1 | 0.1 | 0.0 |  |  |  |
| Retouch Perimeter | Upper | 50 | 0.3 | 1.4 | 0.2 | 0.05 | 50.5 | **0.962** |
|  | Lower | 55 | 0.3 | 0.2 | 0.0 |  |  |  |
| Retouched Edge Curvature | Upper | 50 | 0.3 | 0.4 | 0.1 | 0.51 | 81.8 | **0.613** |
|  | Lower | 54 | 0.2 | 0.8 | 0.1 |  |  |  |
| Kuhn Index | Upper | 255 | 0.1 | 0.3 | 0.0 | 0.00 | 429.6 | **0.999** |
|  | Lower | 187 | 0.1 | 0.2 | 0.0 |  |  |  |
| L:W | Upper | 254 | 1.2 | 0.5 | 0.0 | 1.18 | 403.0 | **0.237** |
|  | Lower | 187 | 1.2 | 0.5 | 0.0 |  |  |  |

References:

Kinnaird, T., Bolòs, J., Turner, A., Turner, S., 2017. Optically-stimulated luminescence
profiling and dating of historic agricultural terraces in Catalonia (Spain). J. Archaeol.
Sci. 78, 66–77.

Munyikwa, k., Kinnaird, T.C., Sanderson, D.C.W, 2021. The potential of portable luminescence readers in geomorphological investigations: a review. Earth Surf. Process. Landforms, 46, 131-150. https://doi.org/10.1002/esp.4975.

Muñoz-Salinas, E., Bishop, P., Sanderson, D.C.W., Zamorano, J.J., 2011. Interpreting luminescence data from a portable OSL reader: three case studies in fluvial settings. Earth Surf. Process. Lanforms 36, 651-660. https://doi.org/10.1002/esp.2084.

Sanderson, D.C.W., Murphy, S. 2010 Using simple portable OSL measurements and laboratory characterisation to help understand complex and heterogeneous sediment sequences for luminescence dating. Quat. Geochronol. 5, 299 – 305.

Stang, D.M., Rhodes, E.J., Heimsath, A.M., 2012. Assessing soil mixing processes and
rates using a portable OSL-IRSL reader: preliminary determinations. Quaternary
Gechronology 10, 314–319.

Stone, A.E.C., Bateman, M.D., Thomas, D.S.G., 2015. Rapid age assessment in the Namib
Sand Sea using portable luminescence reader. Quat. Geochronol. 30, 134–140.
